# Sea-level rise drives unequal erosion of global ecosystem service value

**DOI:** 10.1093/pnasnexus/pgag339

**Published:** 2026-11-03

**Authors:** Hongyu Hu, Yaping Chen, Dong Xu, Yue Zhu, Jiankun He, Qinglong Shao

**Affiliations:** School of Ecology, Sun Yat-sen University—Shenzhen Campus, Shenzhen 518000, China; School of Ecology, Sun Yat-sen University—Shenzhen Campus, Shenzhen 518000, China; Department of Geography, National University of Singapore, Singapore 117570, Singapore; School of Ecology, Sun Yat-sen University—Shenzhen Campus, Shenzhen 518000, China; School of Ecology, Sun Yat-sen University—Shenzhen Campus, Shenzhen 518000, China; School of Ecology, Sun Yat-sen University—Shenzhen Campus, Shenzhen 518000, China

**Keywords:** sea-level rise, ecosystem-service value, climate inequality, shared socioeconomic pathways, natural capital

## Abstract

Sea-level rise (SLR) is rapidly reshaping coastal ecosystems, yet its global implications for ecosystem-service value (ESV) remain poorly quantified. Here, we assess global ESV exposure to SLR under five shared socioeconomic pathways (SSPs), integrating IPCC AR6 projections with detailed valuation modeling over 2030–2100. Across the five SSP-aligned SLR pathways, annual income-adjusted ESV losses attributable to SLR in 2100 range from 130.06 billion Int$2020yr^−1^ under SSP3-7.0 to 339.41 billion Int$2020yr^−1^ under SSP5-8.5. While impervious surface and croplands together account for approximately 59–60% of global absolute losses by 2100 across pathways, wetlands suffer the greatest relative losses (1.21% under SSP1-1.9 vs. 2.20% under SSP5-8.5). Crucially, we reveal a systemic mismatch between historical per-capita carbon responsibility and future per-capita ESV loss. Although absolute losses are concentrated in large countries such as China and the United States, low-responsibility small island and deltaic states face disproportionately high relative declines. This inequity is evident across scenarios: by 2100, the ESV injustice index ranges from 0.450 under SSP3-7.0 to 0.593 under SSP5-8.5, with a value of 0.500 under the intermediate SSP2-4.5 pathway. Our results pinpoint hotspots of natural-capital erosion, providing a vital quantitative basis for adaptation prioritization and loss-and-damage negotiations.

Significance Statement Sea-level rise is a long-term and irreversible consequence of climate change, yet its implications for global natural capital and distributional inequality remain poorly quantified. We provide a global assessment linking projected per-capita ESV losses with historical per-capita carbon responsibility. Our results reveal a structural mismatch between responsibility and loss exposure and introduce an injustice index framework that could be adapted to other climate risks. This work reframes sea-level rise as not only a physical hazard but also a redistribution of natural capital with implications for global sustainability governance.

## Introduction

Sea-level rise (SLR) is one of the most widespread and largely irreversible repercussions of anthropogenic climate change (1–3). Over recent decades, climate-driven SLR has increasingly eroded the ecosystem-service value (ESV) provided by coastal landscapes and adjacent uplands, thereby heightening global socio-ecological risks (4–6). The IPCC Sixth Assessment Report (AR6) projects a global mean sea-level rise of 0.3–1.0 m by 2100 relative to 1995–2014 levels, with higher increases under more intensive emission pathways (7, 8). Observed impacts already include coastal inundation and salinization (9), shoreline erosion (10, 11), and shifts in ecosystem structure and function (7, 12), all of which undermine ecosystem-service provision and human well-being.

Ecosystem services represent the benefits ecosystems provide to society (13, 14). At the global scale, ESV offers a unified accounting framework that links biophysical functions—such as climate regulation, food provision, and water purification—to their economic significance, with aggregate estimates comparable in magnitude to global GDP (15, 16). Framing SLR impacts in ESV terms thus translates ecological degradation into comparable socioeconomic metrics, supporting adaptation and mitigation planning, natural-capital accounting, and ecosystem-informed policy across regions and ecosystems (17).

Although the adverse effects of SLR on coastal ecosystem structure, functioning, and ecosystem services have been well explored from site to regional scales (7, 18, 19), a global framework remains lacking. Existing work has largely focused on individual ecosystem types (e.g. mangroves, salt marshes, or other coastal wetlands) (20, 21) or on risk assessment for a specific country or region (22, 23), whereas an integrated global synthesis is urgently needed, which should span interconnected ecosystems under a range of SLR scenarios—particularly the shared socioeconomic pathways (SSPs). Moreover, key questions remain, including how SLR-induced ESV losses are likely to manifest across countries in absolute and relative terms, and whether projected burdens align with countries’ historical per-capita carbon responsibility. These knowledge gaps limit our ability to identify the ecosystem types and hotspot regions most sensitive to SLR from an ESV-loss perspective and hinder rigorous assessment of prospective global ESV injustice.

To fill the gaps, this study quantifies and maps SLR-attributable ESV losses across multiple ecosystem types and countries under five SSP-aligned SLR pathways from 2030 to 2100, using 2024 as the baseline year for ecosystem areas and unit values. In this context, we pose three logically interconnected research questions: (1) What are the baseline year (2024) spatial patterns of ESV across global ecosystem types and countries? (2) How will global and ecosystem-specific absolute and relative ESV losses attributable to SLR evolve from 2030 to 2100, and which ecosystem types will experience the largest losses? (3) At the national scale, how are SLR-induced ESV losses distributed across countries in absolute and relative terms, and is there a systematic mismatch between countries’ historical per-capita carbon responsibility and their future per-capita ESV losses?

This study makes three contributions. First, we integrate IPCC AR6 regional relative sea-level (RSL) projections with inundation mapping, high-resolution land-cover data, and ESVD v2.0 unit values to establish a harmonized workflow, thereby generating internally consistent and comparable ESV-loss trajectories and hotspot diagnostics across SSPs, years, ecosystems, and countries. Second, by considering all SSP-aligned SLR pathways, we provide a multi-ecosystem global assessment of SLR-attributable ESV loss trajectories from 2030 to 2100, enabling cross-ecosystem comparisons that remain limited in the literature. Third, we quantify the distributional injustice of projected ESV losses relative to historical per-capita carbon responsibility using a concentration curve metric and decompose it into country-level mismatch contributions, connecting ESV losses to climate-justice debates on responsibility, risk sharing, and loss-and-damage.

## Results

### Global patterns of the 2024 ESV baseline

In 2024, all ecosystems considered in this study together provide an annual ESV of approximately 62.23 trillion Int$2020yr^−1^. The resulting spatial pattern is highly uneven across countries, with national ESV totals strongly concentrated in a small number of large-area countries. Notably, Russia stands out as the largest contributor, accounting for 14.6% of global baseline ESV (9.07 trillion Int$2020yr^−1^), followed by China (11%), Brazil (7.4%), Canada (7.2%) and the United States (7.0%). Together, the five countries contribute 47.2% of the global total, forming clear high-ESV clusters in northern Eurasia and the Americas. In contrast, arid regions and countries with limited natural ecosystems—particularly across parts of Africa, the Middle East, and smaller European states—show much lower totals. This pattern reflects the dominant role of land area and forest- and grassland-rich biomes, with boreal, tundra, and tropical forest regions serving as major global ESV reservoirs.

This concentration is further reflected in the top 20 countries (Figure 1b). The leading countries are not only large in area but also contain extensive natural ecosystems whose contributions differ by national context. For example, countries with broad forest domains (e.g. Russia, Canada, and Brazil) show particularly strong forest-driven ESV, whereas others exhibit larger shares from grasslands, croplands, shrublands, or impervious surfaces, highlighting substantial heterogeneity in the ecosystem portfolios underlying similar high national totals.

**Figure 1:**
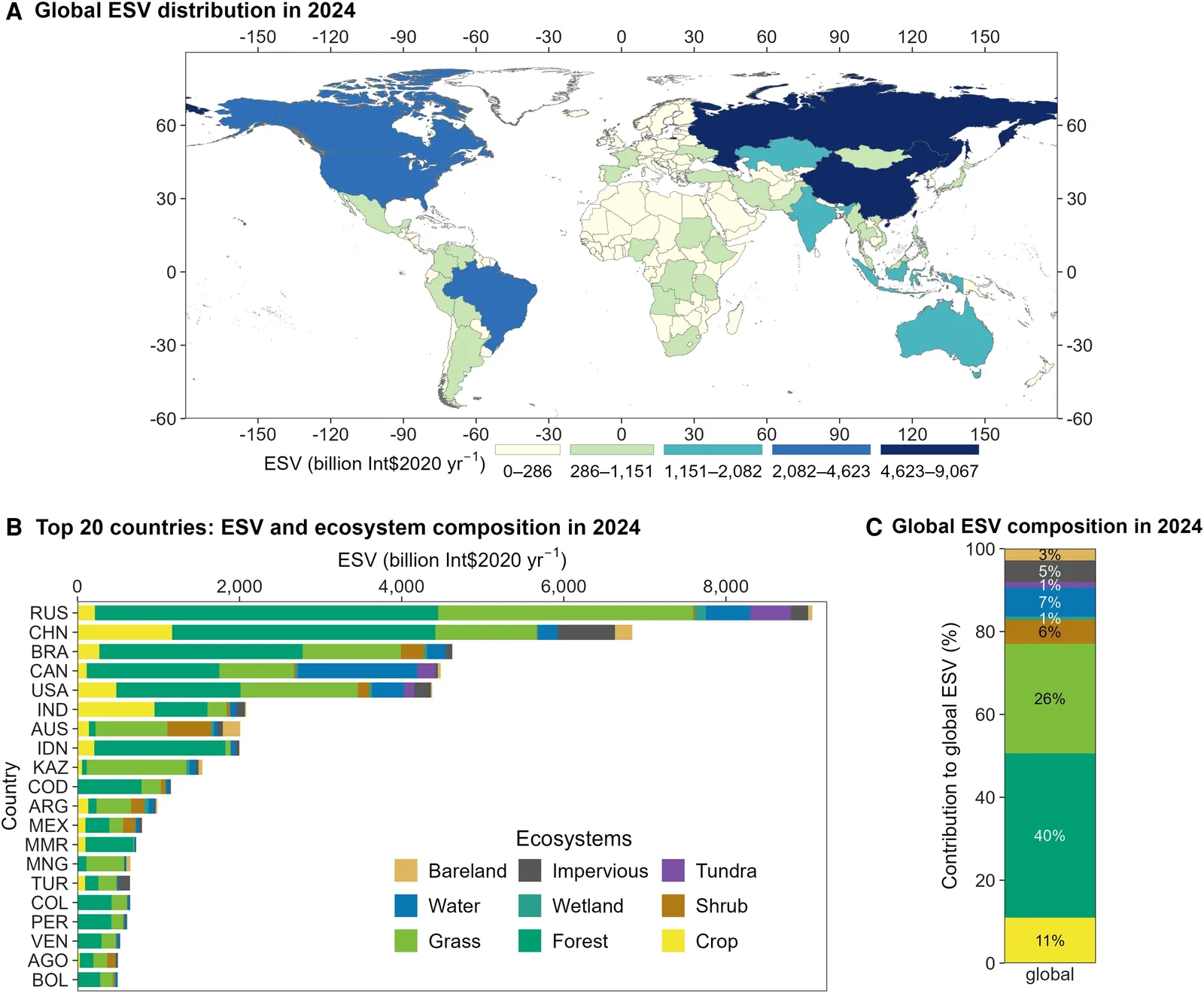
Global baseline distribution and composition of ESV in 2024. (A) National totals of annual ESV (billion Int$2020yr^−1^) for all ecosystems considered in this study; (B) the top 20 countries ranked by national ESV and the composition of their ESV by ecosystem type; and (C) global ESV composition by ecosystem type expressed as the percentage contribution of each type to the global total. The baseline analysis includes the 181 United Nations member states with matched land-cover-area and valuation data. ESV data are from ESVD v2.0 (24).

On the global scale, ESV is also highly uneven across ecosystem types (Figure 1c). Forests contribute 40% of global ESV, representing the largest share, followed by grasslands (26%) and croplands (11%). Water bodies (7%) and shrublands (6%) make moderate contributions, while wetlands (1%) and tundra (1%) contribute smaller but non-negligible shares. Meanwhile, impervious surfaces (5%) and bare land (3%) together make up a notable share of the ESV because ESVD v2.0 assigns these land-cover classes non-zero coefficients; this should be interpreted as an accounting assumption, not as evidence of strong biophysical ecosystem-service provision. Given the dominant contributions of forests and grasslands to global ESV, higher national ESV totals are typically observed in countries with larger forest and grassland areas.

### Projected ESV losses across SLR scenarios

Income-adjusted ESV losses attributable to SLR increase substantially throughout the 21st century across all SSP pathways (Figure 2a). By 2100, global annual losses excluding water range from 130.06 billion Int$2020yr^−1^ under SSP3-7.0 to 339.41 billion Int$2020yr^−1^ under SSP5-8.5. The highest estimate was approximately 2.61 times the lowest; however, this comparison reflects both SSP-specific SLR exposure and SSP-dependent income-adjusted unit values and should not be interpreted as a simple emissions gradient. The corresponding losses were 140.97 billion Int$2020yr^−1^ under SSP1-1.9, 161.01 billion Int$2020yr^−1^ under SSP1-2.6, and 164.46 billion Int$2020yr^−1^ under SSP2-4.5. Interval-based annual loss rates, calculated for decadal intervals over 2030–2100, further reveal pronounced temporal heterogeneity, with a disproportionate share of income-adjusted ESV losses attributable to SLR occurring in the second half of the century (Figure S1). Within each pathway, post-2060 loss rates generally exceed those observed in earlier decades.

**Figure 2:**
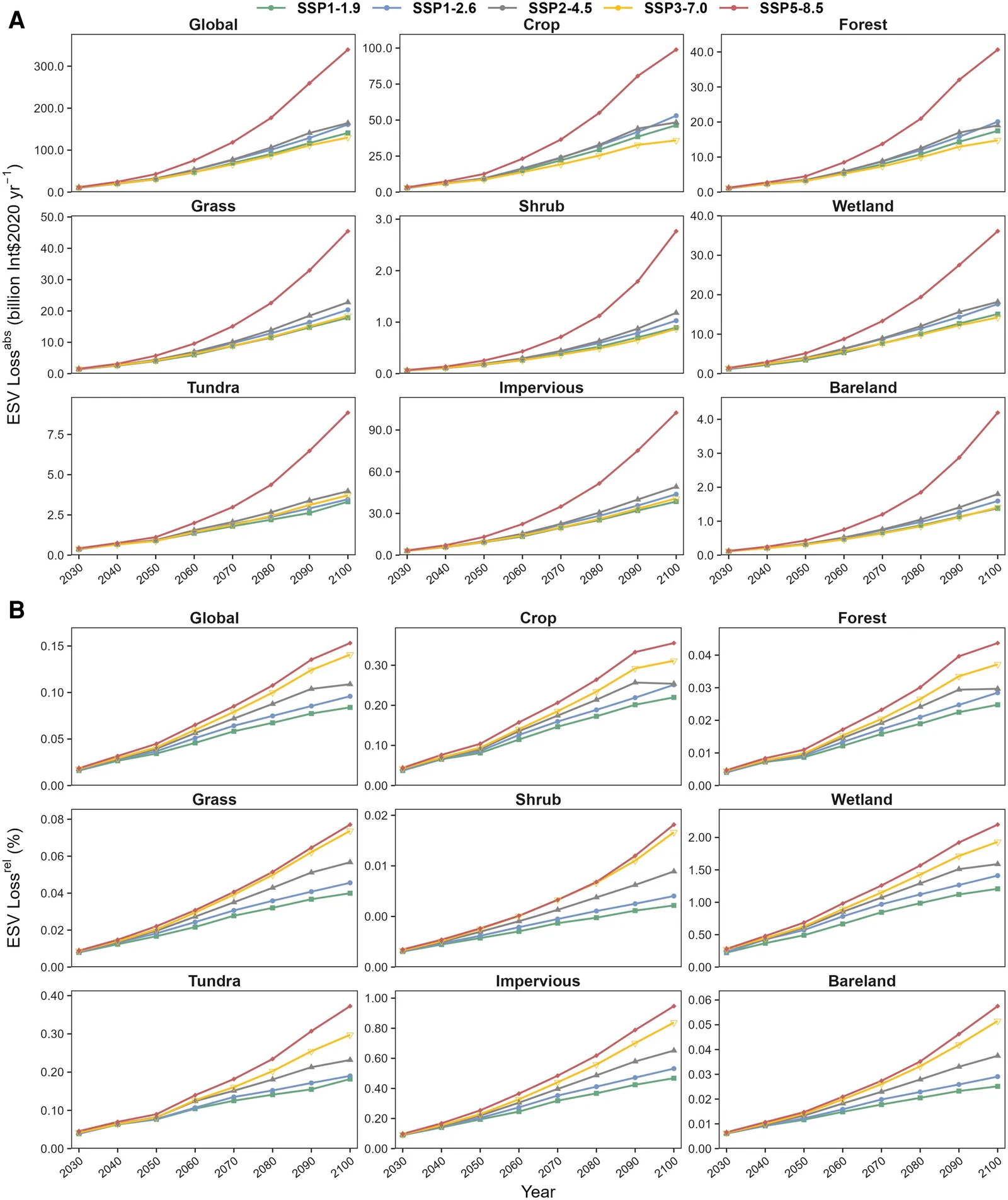
Income-adjusted absolute and relative ESV losses attributable to SLR under five SSP pathways. (A) Annual global and ecosystem-specific absolute ESV losses (Loss^abs^; billion Int$2020yr^−1^) from 2030 to 2100; (B) the corresponding relative ESV losses (Loss^rel^; %), calculated relative to the scenario- and year-specific no-SLR counterfactual ESV. Each panel presents the global non-water total and eight non-water ecosystem types (cropland, forest, grassland, shrubland, wetland, tundra, impervious surface, and bare land) under SSP1-1.9, SSP1-2.6, SSP2-4.5, SSP3-7.0, and SSP5-8.5. Positive values indicate losses. The loss trajectories include the 171 countries with complete SSP GDP-per-capita data. Water is excluded because its apparent ESV gain reflects the reclassification of inundated land as water; the signed nine-class accounting result is shown in Figure S3. ESV coefficients are derived from ESVD v2.0 (24), income trajectories are from SSP Basic Drivers v3.2 (25, 26), and SLR projections are from IPCC AR6 (FACTS: Framework for Assessing Changes To Sea-level) (27–29).

Both absolute and relative ESV losses are widespread across ecosystem types: every non-water land-cover class shows a positive loss (Figures 2a and b). Wetlands exhibit the greatest relative losses, reaching 1.21% under SSP1-1.9 and 2.20% under SSP5-8.5 by 2100 (Figure 2b). Their corresponding absolute losses are 15.09 billion Int$2020yr^−1^ and 36.11 billion Int$2020yr^1^, respectively (Figure 2a). Differences between pathways are especially pronounced for bare land, shrubland, tundra, and impervious surfaces, where absolute losses under SSP5-8.5 are approximately 2.7–3.1 times those under SSP1-1.9 by 2100 (Figure 2a). Linear fits of the global total and ecosystem-specific absolute- and relative-loss trajectories are presented in Figure S2 and summarize the long-term trends shown in Figure 2.

The apparent increase in the ESV of water bodies over time mainly reflects how changes in area are handled: for all non-water ecosystems, portions inundated by SLR are recorded as losses of their corresponding ecosystem area, whereas these inundated areas are simultaneously added to the area of water bodies, thereby producing an accounting gain in water-associated ESV. This accounting gain is represented by a negative water-loss value and does not indicate ecological compensation for losses of non-water ecosystem services. We therefore excluded water from both panels of Figure 2 and from all subsequent national-loss and injustice analyses. For transparency, Figure S3 presents the signed net nine-class accounting result obtained by combining the positive losses across the eight non-water classes with the negative water accounting term. Figure 2b presents the global and ecosystem-specific relative ESV losses attributable to SLR for all five SSP pathways from 2030 to 2100. Relative losses are positive for all eight non-water ecosystem types and increase over time under every pathway. Wetlands consistently exhibit the greatest relative losses, whereas shrublands show the smallest. Under SSP2-4.5 in 2100, relative losses reach 1.59% for wetlands and 0.65% for impervious surfaces, compared with 0.25% for croplands and 0.009% for shrublands.

### Relative losses peak in wetlands and absolute losses in impervious surfaces and croplands

Under SSP2-4.5, relative ESV losses are highly uneven across ecosystem types (Figure 3a). By 2100, relative losses among non-water ecosystems range from 0.009% for shrublands to 1.589% for wetlands. The next-largest relative losses occur in impervious surfaces (0.651%), croplands (0.254%), and tundra (0.232%), followed by grasslands (0.057%), bare land (0.037%), forests (0.030%), and shrublands (0.009%). Wetlands have the largest relative losses at all four selected years, followed by impervious surfaces, whereas shrublands have the smallest (Figure 3a).

**Figure 3:**
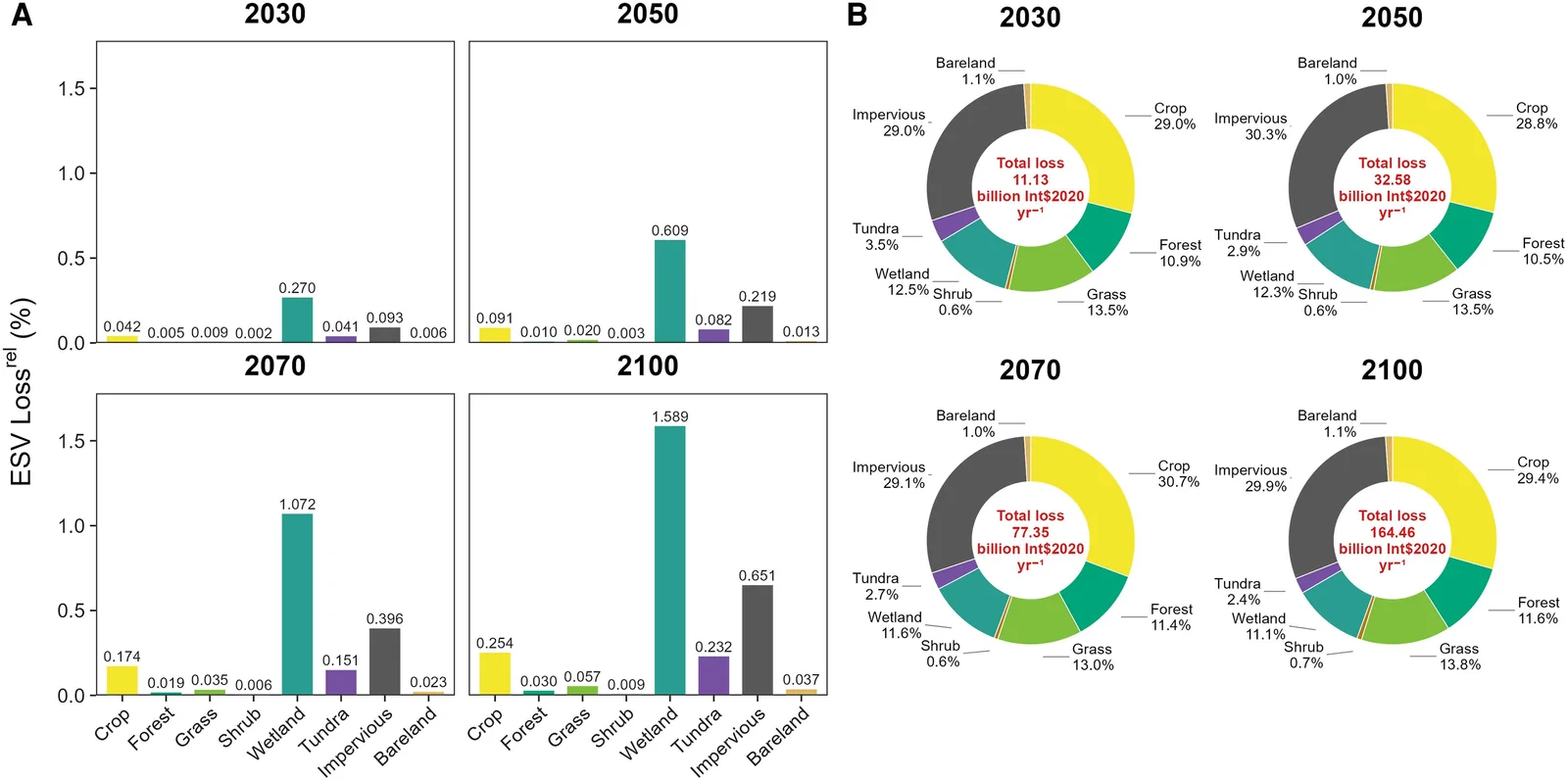
Ecosystem-specific relative ESV losses and absolute-loss composition under SSP2-4.5. (A) Relative ESV loss (Loss^rel^; %) for eight non-water ecosystem types in 2030, 2050, 2070, and 2100, calculated relative to each ecosystem's corresponding no-SLR counterfactual ESV. Numbers above the bars indicate relative loss percentages. (B) Contributions of the same ecosystem types to global absolute ESV loss in the corresponding years. Donut sectors indicate the percentage contribution of each ecosystem type, and the central values indicate the global non-water total loss (billion Int$2020yr^−1^). The eight ecosystem types are cropland, forest, grassland, shrubland, wetland, tundra, impervious surface, and bare land. ESV coefficients are derived from ESVD v2.0 (24), income trajectories are from SSP Basic Drivers v3.2 (25, 26), and SLR projections are from IPCC AR6 (FACTS) (27–29).

The composition of absolute ESV losses varies markedly across ecosystem types (Figure 3b). Across the four selected years, global absolute losses are dominated by impervious surfaces and croplands, together accounting for 58.0–59.8% of total losses; impervious surfaces contribute 29.0–30.3% and croplands contribute 28.8–30.7%. Grasslands, wetlands, and forests also contribute substantially, accounting for 13.0–13.8%, 11.1–12.5%, and 10.5–11.6% of total absolute losses, respectively. Although wetlands experience the largest relative losses, they account for 11.1–12.5% of global absolute losses, while tundra contributes 2.4–3.5%, bare land 1.0–1.1%, and shrublands 0.6–0.7%. Similar patterns persist under other SSP scenarios (Figures S4 and S5), where wetlands consistently show the largest relative losses, but impervious surfaces and croplands dominate absolute losses.

### ESV losses are highly unequal across countries

National ESV losses are highly uneven under the intermediate SSP2-4.5 pathway (Figure 4). National absolute ESV losses are highly concentrated in a small number of countries, led by China (36.66 billion Int$2020yr^−1^), Myanmar (12.57 billion Int$2020yr^−1^), Brazil (11.48 billion Int$2020yr^−1^), Russia (10.68 billion Int$2020yr^−1^), and Viet Nam (7.67 billion Int$2020yr^−1^). The top 20 countries each experience annual losses of at least 1.69 billion Int$2020yr^−1^ by 2100 and together account for 83.3% of the global total, while most other countries experience comparatively small absolute losses.

**Figure 4:**
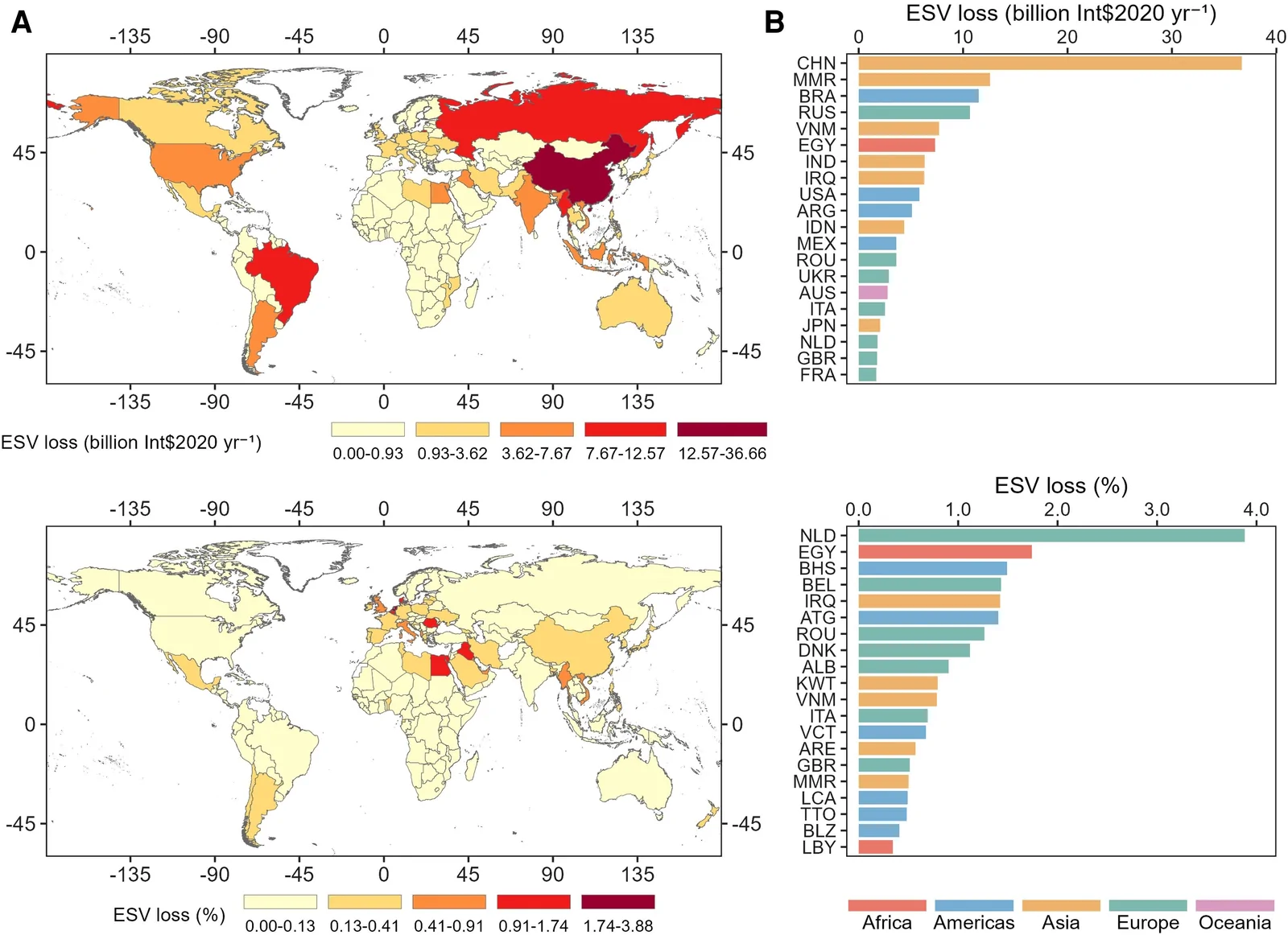
National disparities in absolute and relative ESV losses under the intermediate SSP2-4.5 pathway. (A) Maps of absolute national ESV loss (top; billion Int$2020yr^−1^) and relative ESV loss (bottom; %) in 2100 under SSP2-4.5; and (B) bar charts of the top 20 countries ranked by absolute losses (left) and relative losses (right). ESV coefficients are derived from ESVD v2.0 (24), income trajectories are from SSP Basic Drivers v3.2 (25, 26), and SLR projections are from IPCC AR6 (FACTS) (27–29). Country abbreviations are as follows: CHN (China), MMR (Myanmar), BRA (Brazil), RUS (Russia), VNM (Viet Nam), EGY (Egypt), IND (India), IRQ (Iraq), USA (United States), ARG (Argentina), IDN (Indonesia), MEX (Mexico), ROU (Romania), UKR (Ukraine), AUS (Australia), ITA (Italy), JPN (Japan), NLD (Netherlands), GBR (United Kingdom), FRA (France), BHS (Bahamas), BEL (Belgium), ATG (Antigua and Barbuda), DNK (Denmark), ALB (Albania), KWT (Kuwait), VCT (Saint Vincent and the Grenadines), ARE (United Arab Emirates), LCA (Saint Lucia), TTO (Trinidad and Tobago), BLZ (Belize), and LBY (Libya).

In contrast, the geography of relative loss—measured as the SLR-attributable loss divided by each country's corresponding no-SLR counterfactual ESV—differs markedly. The largest relative losses occur in low-lying delta and coastal lowland nations, including the Netherlands, Egypt, the Bahamas, Belgium, and Iraq. Among the top 20 countries by relative loss, relative losses exceed 0.34% of their corresponding no-SLR counterfactual ESV, approximately three times the global average (0.109%). By comparison, several large countries with substantial absolute losses exhibit only moderate relative losses because of their much larger no-SLR counterfactual ESV stocks. This distinction between absolute and relative loss patterns is also evident in the ecosystem-specific results (Figures S6 and S7). Comparison with the fixed-unit-value estimates shows that income adjustment substantially increases the monetary magnitude of projected losses but has little effect on the identification of national loss hotspots (Figure S9). By 2100, income-adjusted global losses are 57.7–252.5% greater than the corresponding fixed-unit-value estimates across the five pathways, whereas national absolute-loss rankings under SSP2-4.5 remain highly correlated (Spearman's ρ = 0.992), with an identical set of top-20 countries.

### Persistent global ESV injustice and responsibility–loss mismatch

To characterize injustice in future global ESV losses, we define carbon responsibility (Ai) as each country's cumulative CO_2_ emissions up to 2024 normalized by its 2024 population, and use future per-capita ESV loss as the loss indicator (Bi), ensuring consistent per-capita scaling between responsibility and loss. In the Lorenz-like curve ranking countries by increasing Ai (Figure 5a), the distribution lies predominantly above the 1:1 line, indicating that future per-capita ESV losses are disproportionately borne by countries with lower per-capita historical carbon responsibility, revealing a systematic ESV injustice. Under the intermediate SSP2-4.5 pathway, the ESV injustice index increases from 0.401 in 2050 to 0.500 in 2100, indicating a strengthening responsibility–loss mismatch after mid-century.

**Figure 5:**
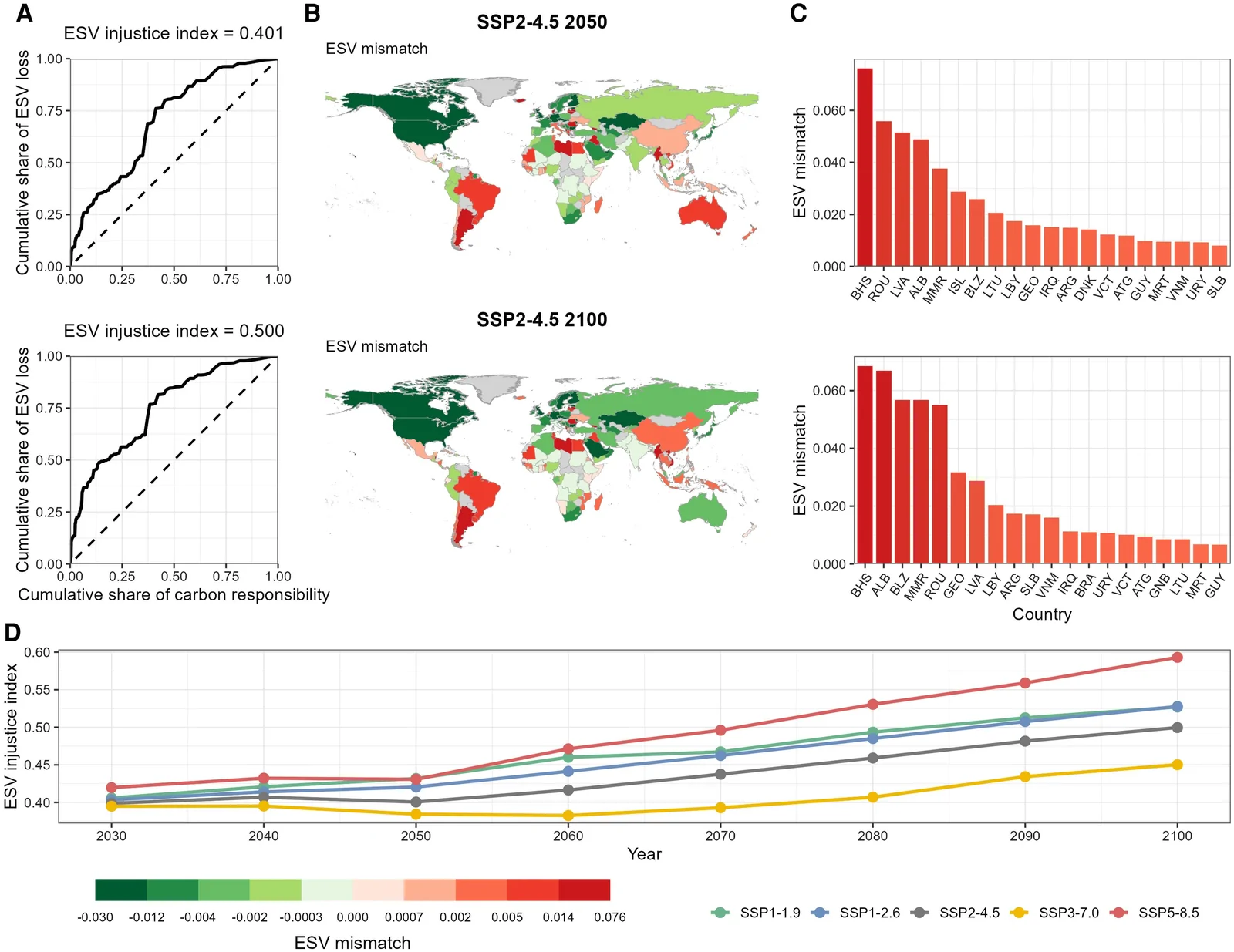
Global injustice of future per-capita ESV losses relative to historical per-capita carbon responsibility (2030–2100). (A) Concentration curves of countries’ cumulative shares of future per-capita ESV losses versus historical per-capita carbon responsibility under SSP2-4.5 in 2050 and 2100. Curves above the equality line indicate losses concentrated in low-responsibility countries; values denote the global ESV injustice index. (B) Global maps of the mismatch index (loss share − responsibility share) under SSP2-4.5 in 2050 and 2100, where red indicates disproportionately higher losses and green indicates lower losses relative to responsibility. (C) Top 20 countries with the largest positive mismatch under SSP2-4.5 in 2050 and 2100. (D) Global ESV injustice index trends across SSPs (2030–2100), showing pathway-specific temporal changes and increasing indices toward the end of the century across all five SSPs. Historical cumulative territorial CO_2_ emissions (1850–2024) are from Our World in Data (30); population in 2024 is from the World Bank (31); SSP-specific GDP-per-capita trajectories are from SSP Basic Drivers v3.2 (OECD ENV-Growth 2025) (25, 26); SSP-specific future population projections are from the SSP Basic Drivers v3.2 (IIASA-WiC POP 2025) hosted by the IIASA SSP Scenario Explorer (32, 33); ESV coefficients are derived from ESVD v2.0 (24); and SLR projections are from IPCC AR6 (FACTS) (27–29).

To identify the spatial sources of this injustice, Figure 5b maps the country-level distribution of ESV mismatch, defined as the difference between each country's loss share and responsibility share. Negative mismatch values occur mainly in countries with relatively high per-capita historical responsibility but lower shares of future per-capita ESV loss, whereas positive values identify countries bearing disproportionately large losses relative to their responsibility. Under SSP2-4.5, this broad spatial contrast persists between 2050 and 2100, although country-level mismatch is reorganized in parts of Europe, Latin America, and Asia.

We identify the main contributors to country-level mismatch by listing the top 20 countries with the largest positive ESV mismatch under SSP2-4.5 in 2050 and 2100 (Figure 5c). Mismatch values are concentrated at the top of the ranking, with a few countries showing substantially larger values than the remainder. The Bahamas records the largest mismatch in both 2050 and 2100, while Romania, Albania, Latvia, and Myanmar remain among the leading countries. Eighteen of the top 20 countries are common to both years. Brazil and Guinea-Bissau enter the top 20 by 2100, replacing Iceland and Denmark. Thus, the late-century increase in the global injustice index is accompanied by moderate, rather than wholesale, reorganization of the principal national mismatch hotspots.

At the global scale, the ESV injustice index increases toward the end of the century across all five SSP pathways, although the temporal trajectories and ordering of the pathways differ (Figure 5d). Under SSP1-1.9, the index increases from 0.406 in 2030 to 0.527 in 2100, whereas under SSP5-8.5 it increases from 0.420 to 0.593 over the same period. Temporary mid-century declines occur under SSP2-4.5, SSP3-7.0, and SSP5-8.5, whereas the indices under SSP1-1.9 and SSP1-2.6 increase throughout the study period. By 2100, the index ranges from 0.450 under SSP3-7.0 to 0.593 under SSP5-8.5, with SSP2-4.5 reaching 0.500. The non-monotonic ordering across pathways indicates that the responsibility–loss mismatch reflects the combined effects of SLR exposure, population trajectories, and national ESV-loss patterns, rather than emission forcing alone.

## Discussion

SLR-driven ESV losses exhibit strong temporal accumulation and path dependence. Even under stringent mitigation, sea level continues to rise due to ocean thermal inertia and delayed ice-sheet responses, implying that a portion of natural-capital erosion is already committed (34). Emissions mitigation can reduce long-term physical SLR exposure, but the monetary ordering of losses across the integrated SSP pathways is also shaped by pathway-specific income trajectories. Non-linear coastal hypsometry further amplifies divergence across emission pathways in the second half of the century (10), as incremental sea-level rise can trigger disproportionate expansions of inundated areas (35). The widening spread in projected monetary losses across scenarios therefore reflects the combined effects of SSP-specific SLR exposure and SSP-dependent income-adjusted unit values and should not be attributed to differences in SLR magnitude alone. Compared with GDP- or asset-based metrics, the ESV perspective captures changes in annual ecosystem-service flows (Int$2020yr^−1^). These flows are generated by underlying natural-capital stocks, represented here by the extent and ecological capacity of ecosystems. SLR-induced inundation reduces the mapped ecosystem area and consequently diminishes the annual flow of ecosystem services. Our analysis monetizes changes in these annual service flows rather than independently estimating the monetary asset value of the underlying ecosystem stocks, consistent with ecosystem-accounting approaches and broader planetary-boundary perspectives on ecological limits (36–39). Although emissions mitigation can limit long-term physical SLR exposure, a residual component remains because of committed sea-level rise, underscoring the need to couple emissions reduction with sustained adaptation and coastal ecosystem protection (1, 40).

Cross-ecosystem comparisons reveal a fundamental distinction between relative vulnerability and absolute loss attribution. Wetlands experience the largest proportional declines because of their concentration in low-lying zones and sensitivity to elevation thresholds (12), including risks of coastal squeeze (41–44). Yet impervious surfaces and croplands dominate global absolute ESV losses because of their substantial no-SLR counterfactual ESV stocks and spatial overlap with exposed coastal areas. Thus, the ecosystem most vulnerable in relative terms is not necessarily the largest contributor to aggregate loss. Relative impacts primarily reflect exposure sensitivity and threshold dynamics, whereas absolute losses are governed by the distribution of no-SLR counterfactual ESV stocks. Although the aggregation of wetland classes may render absolute wetland losses conservative, the conclusion that wetlands experience the greatest relative losses remains robust. Recognizing this accounting distinction is essential for ecosystem-specific risk interpretation and policy prioritization.

At the national scale, hotspots of absolute loss diverge systematically from hotspots of relative loss normalized by each country's corresponding no-SLR counterfactual ESV. Absolute losses reflect the scale of exposed natural-capital stocks, whereas relative losses capture shock intensity relative to national endowments. Reliance on aggregate loss levels alone therefore risks overlooking countries facing severe proportional shocks and resilience pressures (45). Beyond distributional inequality lies a normative dimension: persistent misalignment between historical per-capita carbon responsibility and future per-capita ESV losses reveals a structural responsibility–loss mismatch (46, 47). This mismatch is spatially clustered and evolves differently across pathways. Across all five pathways, the index increases toward the end of the century, with SSP5-8.5 showing the strongest late-century increase; however, the non-monotonic ordering across pathways indicates that differences in injustice reflect the combined effects of SLR exposure, population trajectories, and national loss patterns rather than emission forcing alone. Framed in this way, the mismatch metric provides a distribution-sensitive diagnostic relevant to the international climate-policy agenda on Loss and Damage by translating SLR-driven natural-capital erosion into a comparable cross-country indicator (48, 49). However, the mismatch metric should complement rather than replace measures of vulnerability, adaptive capacity, and socioeconomic constraints, because these indicators serve different policy functions. Absolute ESV losses indicate the overall scale of natural capital at risk, relative losses capture the severity of impacts on national ecosystem-service endowments, and the responsibility–loss mismatch adds a distributional-justice perspective. Considered together, these dimensions can support more transparent prioritization of adaptation and Loss and Damage finance, particularly for countries facing high relative losses despite low historical responsibility (50, 51). More broadly, jointly considering ecological limits and distributional consequences is consistent with emerging efforts to define Earth-system boundaries that are both safe for ecological stability and just for people (52).

Our results also have implications for the Sustainable Development Goals (SDGs) and the Kunming–Montreal Global Biodiversity Framework (GBF). The spatial identification of ecosystems and countries facing substantial SLR-induced ESV losses may help integrate ecosystem-service risks into climate adaptation planning (SDG 13) and inform the conservation and restoration of coastal, terrestrial, and wetland ecosystems (SDGs 14 and 15) (53). In relation to the GBF, these spatial results may inform the prioritization of ecosystem restoration and conservation actions (54), consistent with its targets on biodiversity-inclusive spatial planning, ecosystem restoration, area-based conservation, climate resilience, and the maintenance of nature's contributions to people (55), particularly in coastal areas where projected losses are high relative to national ecosystem-service endowments. The responsibility–loss mismatch further provides a comparative indicator for identifying countries that may warrant greater consideration in international adaptation and Loss and Damage support (48, 49). However, the standardized ESV estimates are intended for comparative screening and priority identification, rather than for directly determining compensation amounts or site-specific management actions.

A limitation of our valuation framework is that the SSP-dependent income adjustment is implemented at the national scale using a common income elasticity of 0.59 for all country–ecosystem coefficients, based on global evidence on willingness to pay for public natural capital (56). Although this enables the main analysis to reflect scenario-dependent changes in real GDP per capita, it does not capture within-country heterogeneity in beneficiaries, local ecosystem scarcity, or service-specific socioeconomic responses. More broadly, ecosystem-service delivery can vary substantially within broad land-cover classes because of management practices and landscape composition and configuration, producing nonlinear responses and trade-offs among services (57, 58). Cultural ecosystem services are especially context dependent, and species richness alone does not reliably predict their perceived value (59). Implementing such refinements consistently would require harmonized spatially explicit and service-specific empirical relationships across all countries and ecosystem types, which are beyond the current coverage of ESVD v2.0 (60). Moreover, embedding income trajectories in unit ESVs means that cross-scenario differences in monetary losses reflect both physical SLR exposure and socioeconomic valuation changes, complicating their attribution to SLR magnitude alone and the interpretation of the responsibility–loss mismatch. We therefore use the income-adjusted estimates as the main scenario-dependent results and report the fixed-unit-value estimates as a comparison that holds 2024 unit values constant (Figure S9). The close agreement in national loss rankings between the two valuation treatments supports the robustness of the main geographical patterns; however, the absolute monetary estimates should be interpreted as scenario-dependent valuation results rather than direct measures of welfare loss or compensation requirements.

## Conclusion

This study provides a global, multi-ecosystem assessment of SLR-driven ESV losses using IPCC AR6 projections with high-resolution land-cover and valuation data. Our results show that SLR represents a long-term and path-dependent erosion of natural capital that continues even under strong mitigation, reflecting the committed nature of sea-level change. Across all SSP pathways, income-adjusted ESV losses attributable to SLR increase throughout the twenty-first century, with a growing share of losses occurring in the latter half of the century and substantial differences emerging across ecosystems and regions. Wetlands experience the greatest relative losses, highlighting their sensitivity to inundation thresholds, while impervious surfaces and croplands account for the largest share of absolute losses because of their large no-SLR ESV stocks and spatial exposure. At the national scale, a small number of countries with large exposed ESV stocks dominate total losses, whereas low-lying small states and deltaic countries experience the most severe relative losses, underscoring the uneven geography of SLR impacts. Linking future per-capita ESV losses to historical per-capita CO_2_ responsibility reveals a persistent responsibility–loss mismatch, with countries having the lowest historical per-capita carbon responsibility facing disproportionately large shares of future per-capita ESV loss. The mismatch index increases toward the end of the century across all five pathways, with the strongest late-century increase occurring under SSP5-8.5; however, its non-monotonic ordering across pathways indicates that the magnitude of injustice reflects combined physical and socioeconomic effects rather than emission forcing alone. Our findings highlight that mitigation can limit long-term physical SLR exposure, but both the monetary magnitude and distribution of ESV losses depend jointly on SLR and SSP-specific socioeconomic trajectories. Integrating ESV considerations into climate risk assessments can help bridge physical impacts with socioeconomic consequences, supporting more equitable and ecosystem-informed responses to rising seas and informing progress toward the SDGs and the Kunming–Montreal GBF. More broadly, rising seas should be understood not only as a physical hazard but as a long-term redistribution of natural capital under climate change, reshaping the geography of ecological risk and responsibility across the century.

## Materials and methods

### Land-cover data and ecosystem classification

Global land-cover data was derived from the 10-m global land-cover product developed by Gong et al. (61, 62), based on a consistent classification scheme; we used the version updated on September 20, 2024. This original product distinguishes 10 land-cover classes, including cropland, forest, grassland, shrubland, wetland, water, tundra, impervious surface, bare land and snow/ice. For valuation purposes, we used only nine land-cover classes, excluding snow/ice, because reliable ESV data were not available for this class.

To link land cover with ecosystem-service valuation, we constructed a crosswalk between the land-cover legend and the biome–ecosystem classification adopted by ESVD v2.0 (24), which is based on the FAO Global Ecological Zoning (63) and the IUCN Global Ecosystem Typology (64, 65) (https://www.esvd.info/classifications). We did not distinguish finer subclasses at the land-cover level. Instead, we treated the nine land-cover types described above as our target ecosystem classes and aggregated the biome–ecosystem types in ESVD 2.0 into these nine classes according to their dominant characteristics. For example, all ESVD ecosystem types related to forests were grouped into the “forest” class; types associated with intensive agriculture or plantations were grouped into “cropland”; types associated with rangelands and natural grasslands were grouped into “grassland”; both inland and coastal-wetland types were grouped into “wetland”; and urban blue–green infrastructure and related artificial surface features were mapped to “impervious surface.” In this crosswalk, the impervious surface class captures ecosystem-service values associated with urban blue–green infrastructure and other nature-based features embedded within built environments, rather than assigning ESV to sealed surfaces themselves (66). In this way, we derived an ecosystem classification that is consistent with the nine land-cover types while retaining as much information as possible from the ESVD classification. All subsequent analyses of ecosystem spatial distribution and ESV are based on this harmonized nine-class ecosystem typology; the detailed crosswalk is provided in Table S1.

For wetlands, ESVD 2.0 distinguishes several ecosystem types, including coastal systems and inland wetlands, and some coastal-wetland subtypes exhibit substantially higher unit values than most inland wetland types (16, 60, 67). However, the 10-class land-cover product used in this study provides only a single “wetland” category and does not allow a reliable global separation of coastal and inland wetlands at our working resolution. To implement a consistent multi-ecosystem framework for global ESV estimation, we therefore adopted a unified wetland unit value derived from aggregated statistics across all wetland-related ecosystem types in ESVD 2.0. This treatment does not fully capture the higher unit values of coastal wetlands and thus renders our wetland-related loss estimates conservative. Nevertheless, given the spatial resolution of the land-cover data and the overall level of model uncertainty, this approximation represents a reasonable compromise for characterizing global ESV losses and their broad spatial patterns across ecosystem types under sea-level rise scenarios.

### Ecosystem-service valuation

ESV data were obtained from the ESVD 2.0 database (https://www.esvd.info) (24). We used the standardized monetary-value field provided by ESVD 2.0, expressed in international dollars per hectare per year at 2020 price levels (Int$2020 ha^−1^ yr^−1^) (60). The classification of ecosystem services follows the framework proposed by TEEB, which is consistent with the Millennium Ecosystem Assessment, and distinguishes four main categories and 23 subcategories of services: provisioning (food, water, raw materials, genetic resources, medicinal resources and ornamental resources); regulating (air-quality regulation, climate regulation, moderation of extreme events, regulation of water flows, waste treatment, erosion prevention, maintenance of soil fertility, pollination and biological control); habitat (maintenance of life cycles and maintenance of genetic diversity); and cultural (aesthetic information, opportunities for recreation and tourism, inspiration for culture, art and design, spiritual experience, information for cognitive development, and existence and bequest values).

Based on ESVD 2.0, we compiled eligible unit value observations (Int$2020 ha^−1^ yr^−1^) for each harmonized ecosystem class and service type (68). For country–ecosystem-service combinations with at least five eligible country-specific valuation observations, we used the corresponding country-level mean. Otherwise, we applied the continental mean and, where this was unavailable, the global mean. If no eligible observation was available globally for a given ecosystem–service combination, that service component was assigned a value of zero for the corresponding ecosystem class. To avoid double counting and methodological inconsistencies, we retained original estimates that valued a single service and whose biome or ecozone codes mapped unambiguously to a single harmonized ecosystem class. Valuations that bundled multiple services, spanned codes assigned to different harmonized ecosystem classes, or were derived purely through value transfer were excluded from the summary statistics (60).

To reduce the influence of extreme values that might distort the summary statistics, we adapted the two-step outlier screening approach of Brander et al (60). First, within each harmonized ecosystem class–service combination, statistical outliers were removed from the unit values using the 1.5 × interquartile range (IQR) rule. Second, within each harmonized ecosystem class–service combination, remaining extreme observations were examined manually in light of their biophysical and socioeconomic context, and highly unrepresentative cases were excluded. For each combination, the final unit ESV was calculated as the mean of the valuation observations after outlier screening. The resulting unit values are broadly consistent in magnitude and distribution with those reported in previous global syntheses (60), indicating an overall coherence of the valuation patterns. The spatial distribution and composition of unit ESV coefficients for the nine ecosystem types are shown in Figure S8.

For a given ecosystem type i in year t, ESV was calculated as


(1) $$ESV_{i,t}=A_{i,t}\times V_{i}$$

where $A_{i,t}$ is the area of ecosystem type i in year t (ha), and $V_{i}$ is the unit ESV for that ecosystem type (Int$2020 ha^−1^ yr^−1^). Global or national totals were obtained by summing ESV across all ecosystem types.

For the main analysis, we allowed country-level unit ESVs to vary over time according to SSP-dependent changes in real GDP per capita. GDP-per-capita trajectories were obtained from SSP Basic Drivers v3.2 (May 2025), using the OECD ENV-Growth 2025 projections for GDP per capita at purchasing-power parity (constant 2017 USD person^−1^ yr^−1^) (25, 26). Country-level GDP per capita in 2024 was log-linearly interpolated between the 2020 and 2025 Historical Reference values. For country *c*, scenario *s*, and year *t*, the income-adjustment factor was calculated as


(2) $$IF_{c,s,t}={\left(\frac{GDPpc_{c,s,t}}{GDPpc_{c,2024}}\right)}^{0.59}$$

where 0.59 is the estimated income elasticity of willingness to pay for public natural capital (56). The corresponding income-adjusted unit ESV was calculated as:


(3) $$V_{c,i,s,t}=V_{c,i,2024}\times IF_{c,s,t}$$

where $V_{c,i,2024}$ is the 2024 unit ESV of ecosystem type *i* in country *c*. The adjustment factor equals 1 in 2024. The matched 2024 land-cover-area and valuation data covered 181 United Nations member states. Missing SSP GDP-per-capita values were not imputed; 171 of these countries had complete income trajectories and formed the consistent sample used for the income-adjusted projection, loss, and fixed-unit-value comparison analyses.

The processed country–ecosystem unit values, service-specific components, geographic source levels, and the numbers of underlying valuation observations and unique primary studies are provided in Dataset S1.

### Sea-level rise scenarios and coastal inundation

We used five SLR scenarios covering the range of global SLR as projected by the IPCC AR6 (34). The five scenarios correspond to SSP1-1.9, SSP1-2.6, SSP2-4.5, SSP3-7.0 and SSP5-8.5. We did not include SSP4 (e.g. SSP4-6.0) because the five selected scenarios already cover the full spectrum from the strongest mitigation pathway (SSP1-1.9) to a high-end emissions pathway (SSP5-8.5). In comparison, SSP4 places greater emphasis on the socioeconomic dimension of North–South disparities and domestic inequality, while its medium- to high-emission characteristics overlap with those of SSP2-4.5 and SSP3-7.0. Therefore, SSP4 was excluded from this study. We selected SSP2-4.5 as the focal pathway for detailed presentation because it represents a middle-of-the-road future with intermediate socioeconomic development and radiative forcing, thereby providing a balanced case between strong-mitigation and high-emission pathways. Its use as a representative moderate pathway is also consistent with recent assessments of coastal-wetland vulnerability, coastal flood losses, and ecosystem-service-related climate impacts (69–71). Results across all five pathways are presented in Figure 2, while the corresponding ecosystem-specific results for the other four pathways are provided in Figures S4 and S5. SSP5-8.5 was retained only as a high-end pathway for comparison and was not treated as a baseline, business-as-usual, or most-likely pathway.

Regional RSL projections were taken from the IPCC AR6 Sea Level Projections dataset, which is generated with the Framework for Assessing Changes to Sea level (FACTS) v1.0 (27–29). This dataset combines contributions from ocean thermal expansion, glaciers, ice sheets, land-water storage and glacial isostatic adjustment, and accounts for gravitational, rotational and solid-Earth “fingerprint” effects. For each coastal grid cell we extracted RSL projections for multiple time slices between 2024 and 2100, expressed relative to the modern baseline period used in AR6.

Coastal inundation was simulated by combining these RSL projections with the Shuttle Radar Topography Mission (SRTM) digital elevation model (72), following the approach of Schuerch et al (7). First, SRTM elevation data were intersected with a global coastline mask to delineate the coastal floodplain. For each scenario and time slice, the projected RSL was added to a reference tidal level to obtain the projected water level, and all land cells with elevations below this level were classified as inundated. This procedure produced, for each scenario-year combination, a global inundation raster representing the potential SLR-induced inundation extent in the absence of additional coastal protection, adaptation measures or geomorphic feedbacks.

The inundation layers were then overlaid with the 10-m global land-cover data to quantify the area of each ecosystem type that becomes inundated under each scenario and time slice. For non-water ecosystems (cropland, forest, grassland, shrubland, wetland, tundra, impervious surface and bare land), inundated areas were treated as losses of that ecosystem's area. In intermediate products, inundated terrestrial cells were reclassified as “water,” resulting in an apparent increase in water area and its associated ESV over time. Because this apparent gain represents land-cover reclassification rather than ecological compensation for lost non-water ecosystem services, water was excluded from both panels of Figure 2 and from all subsequent ecosystem-specific, national-loss, and injustice analyses. For transparency, the signed net nine-class accounting result, obtained by combining positive losses across the eight non-water classes with the negative water accounting term, is reported separately in Figure S3.

For computational efficiency and to harmonize spatial resolution across datasets, all inundation outputs and land-cover data were resampled to a common 200-m grid prior to area and ESV calculations. Accordingly, only ecosystem areas located within the projected coastal inundation extent contributed to the estimated ESV losses. Unaffected inland grid cells did not contribute to the loss estimates. Country-level losses were obtained by aggregating the affected coastal grid cells within each national boundary.

### Calculation of ESV losses under SLR scenarios

For each scenario and time slice, we first updated the ecosystem type of each grid cell on a common 200-m grid. Within each country, the total number of grid cells and the total area represented in the analysis were kept constant across all scenarios and years. As sea level rises, terrestrial cells within the projected inundation extent are reclassified from their original ecosystem (e.g. forest, cropland) to “water”. Consequently, the number and area of grid cells assigned to non-water ecosystem types decrease, while those assigned to water increase. The total number of grid cells and the total area represented within each national boundary remain unchanged. We then aggregated, at the national or global scale, the number of grid cells of each ecosystem type and multiplied by the grid-cell area to obtain the area of each ecosystem type under each scenario and at each time slice.

Using the projected ecosystem areas and the income-adjusted unit ESVs defined above, we constructed a no-SLR counterfactual and a corresponding with-SLR estimate for each country, ecosystem type, SSP, and future year. The no-SLR counterfactual retained the 2024 ecosystem area while allowing the unit ESV to vary according to the scenario- and year-specific income-adjustment factor:


(4) $$ESV_{c,i,s,t}^{noSLR}=A_{c,i,2024}\times V_{c,i,s,t}$$

where *A_c,i,2024_* is the 2024 area of ecosystem type *i* in country *c*, and *V_c,i,s,t_* is its income-adjusted unit ESV under scenario *s* in year *t*. The with-SLR ESV was calculated using the projected ecosystem area after SLR-induced inundation:


(5) $$ESV_{c,i,s,t}^{SLR}=A_{c,i,s,t}\times V_{c,i,s,t}$$

where *A_c,i,s,t_* is the projected area of ecosystem type *i* under scenario *s* in year *t*. The same income-adjusted unit ESV was applied to the no-SLR and with-SLR states, ensuring that their difference reflects only the SLR-induced change in ecosystem area.


(6) $$Loss_{c,i,s,t}^{abs}=ESV_{c,i,s,t}^{noSLR}-ESV_{c,i,s,t}^{SLR}$$

Relative ESV loss was calculated by normalizing the absolute loss by the corresponding no-SLR counterfactual ESV:


(7) $$Loss_{c,i,s,t}^{rel}=\frac{ESV_{c,i,s,t}^{noSLR}-ESV_{c,i,s,t}^{SLR}}{ESV_{c,i,s,t}^{noSLR}}\times 100\text{\%}$$

Thus, $Loss_{c,i,s,t}^{rel}$ is positive when SLR reduces ESV.

Country-level, ecosystem-level, and global absolute ESV losses were obtained by summing the corresponding monetary losses before calculating aggregate relative losses. Water was excluded from global loss totals and from all relative-loss, national-loss, and injustice analyses; the signed net nine-class accounting result was reported separately in Figure S3. All monetary ESV estimates are expressed in international dollars at 2020 price levels (Int$2020yr^−1^).

For comparison with the main income-adjusted analysis, we repeated the loss calculations while holding each country–ecosystem unit ESV at its 2024 value in all future years. The fixed-unit-value calculation used the same projected ecosystem areas, no-SLR and with-SLR definitions, water-exclusion rule, and 171-country sample as the main analysis. The comparison between the income-adjusted and fixed-unit-value results is reported in Figure S9.

To summarize temporal trends, we fitted ordinary least-squares linear regressions separately to each loss series using the eight decadal observations from 2030 to 2100. In Figures S2 and S3, the fitted lines are shown with 95% confidence bands. Figure S2 presents fits for absolute and relative losses of the global non-water total and the eight non-water ecosystem classes under each of the five SSP pathways. Figure S3 presents the calculated trajectories and corresponding linear fits for the signed net nine-class accounting result under each pathway. Analogous country-level regressions were fitted for national absolute and relative ESV losses. The slope, intercept, fitted equation centered at 2030, coefficient of determination (R^2^), root-mean-square error (RMSE), and number of observations are provided in Dataset S2.

### Measuring injustice between historical carbon responsibility and future ESV losses

To assess the global injustice of future ESV losses under sea-level rise scenarios, we assembled data for 193 United Nations member states and compared countries’ historical per-capita carbon responsibility with future per-capita ESV losses. Historical cumulative territorial CO_2_ emissions were obtained from Our World in Data (30). We used cumulative emissions reported through 2024; 2024 values were unavailable for two countries, which remained missing and were handled through the complete-case criteria described below. Country population in 2024 was sourced from the World Bank (31), while scenario-specific future population projections were obtained from the updated SSP population and human capital projections (version 3.2) developed by IIASA and the Wittgenstein Centre (32, 33). For each scenario-year combination, the injustice and mismatch indices were calculated using countries with non-missing historical per-capita responsibility, SSP population, and income-adjusted ESV loss, and with both positive responsibility and positive loss. Depending on the scenario and year, 145–151 countries met these criteria.

On this basis, we constructed a global ESV injustice indicator. We first defined per-capita carbon responsibility and per-capita ESV loss as:


(8) $$A_{i}=\frac{CumCO_{2}^{i,\;\leq 2024}}{Pop_{i,2024}}$$

(9) $$B_{i,s,t}=\frac{ESVloss_{i,s,t}}{Pop_{i,s,t}}$$

Here, *A_i_* denotes the historical per-capita carbon responsibility of country *i*; $CumCO_{2}^{i,\;\leq 2024}$ is cumulative territorial CO_2_ emissions up to 2024, and $Pop_{i,2024}$ is the country's population in 2024. $B_{i,s,t}$ denotes the per-capita ESV loss of country *i* in year *t* under scenario *s*; $ESVloss_{i,s,t}$ is the scenario- and year-specific ESV loss, and $Pop_{i,s,t}$ is the corresponding scenario-year population. Countries were then ranked from low to high *A_i_* to construct a Lorenz-like concentration curve, plotting cumulative responsibility shares on the x-axis and cumulative loss shares on the y-axis. We quantified the global responsibility–loss mismatch by the area between the concentration curve and the 45° equality line; for ease of interpretation, we define ESV injustice as the negative of the concentration index (CI), such that larger positive values indicate a stronger concentration of future per-capita ESV losses among low-responsibility countries (i.e. greater global injustice).

Let *R* denote cumulative responsibility share and $L_{s,t}(R)$ the corresponding cumulative loss share after countries are ranked in ascending order of *A_i_*. The global ESV injustice index was calculated as


(10) $$Injustice_{s,t}=2\int_{0}^{1}L_{s,t}(R)dR-1$$

Positive values indicate that future per-capita ESV losses are disproportionately concentrated among countries with lower historical per-capita carbon responsibility.

Building on the global metric, we further constructed a country-level mismatch index to identify major contributors and spatial sources. We first defined each country's responsibility share and loss share, and then computed the mismatch index as


(11) $$share_{i,s,t}(A)=\frac{A_{i}}{\sum_{\;j\in \Omega_{s,t}}\;A_{j}}$$

(12) $$share_{i,s,t}(B)=\frac{B_{i,s,t}}{\sum_{\;j\in \Omega_{s,t}}\;B_{\;j,s,t}}$$

(13) $$mismatch_{i,s,t}=share_{i,s,t}(B)-share_{i,s,t}(A)$$

Here, $\Omega_{s,t}$ denotes the set of countries satisfying the complete-case criteria for scenario *s* and year *t*, and *j* indexes countries within this set. $share_{i,s,t}(A)$ and $share_{i,s,t}(B)$ denote country *i*'s shares of the summed country-level per-capita responsibility and per-capita ESV loss measures, respectively. A positive *mismatch_i,s,t_*indicates that a country bears a disproportionately large share of per-capita ESV loss relative to its historical per-capita responsibility (a disadvantaged loss position), whereas a negative value indicates a net-responsibility position.

## Supplementary Material

pgag339_Supplementary_Data

## Data Availability

The complete analysis and plotting code, processed valuation data, and derived output datasets supporting all figures and tables are publicly available in the Zenodo repository *Processed datasets and reproducibility workflow for global ecosystem-service value losses under sea-level rise* (https://doi.org/10.5281/zenodo.21912761). Raw third-party source datasets are not redistributed because they remain subject to their original access and licensing conditions. The processed subsets and author-generated summaries included for reproducibility, together with the sources, versions, access information, and required workflow locations of the original datasets, are fully documented in the archived materials.
