# Supplementary material for: Sea-level rise drives unequal erosion of global ecosystem service value
Source: PNAS Nexus. 2026 Nov 3;5(11):pgag339. doi: 10.1093/pnasnexus/pgag339 (PMC13729132; doi:10.1093/pnasnexus/pgag339)
Supplement: Supplementary file 1 [file pgag339_supplementary_data.zip › pgag339_Supplementary_Data.pdf]

PNAS nexus

**Supplementary Information for**
Sea-level rise drives unequal erosion of global ecosystem service value.

Hongyu Hu, Yaping Chen, Dong Xu, Yue Zhu, Jiankun He, Qinglong Shao*.

*Qinglong Shao
Email: shaoqlong@mail.sysu.edu.cn

**This PDF file includes:**

Supplementary text
Figures S1 to S9
Table S1
Legends for Datasets S1 to S2
SI References

## Supplementary Information Text

### Global income-adjusted ESV loss trajectories and interval-based increases

Figure S1 shows the mean annual increase in income-adjusted absolute ecosystem service value (ESV) loss attributable to SLR over successive decadal intervals from 2030 to 2100. Rates generally increase toward the end of the century but are not strictly monotonic. Across all SSPs, increases after 2050 exceed those during 2030–2050, while SSP5-8.5 shows especially rapid late-century growth, peaking at 8.26 billion Int$2020 $yr^{-2}$ during 2080–2090. The 2024–2030 interval is excluded because its six-year duration is not directly comparable with the successive ten-year intervals.

Figure S2 presents ordinary least-squares linear fits to the income-adjusted absolute and relative ESV loss trajectories shown in Fig. 2. Panel (a) shows absolute losses and panel (b) shows relative losses for the global non-water total and eight non-water ecosystem classes under all five SSP pathways, using eight decadal observations from 2030 to 2100. All fitted slopes are positive, indicating increasing SLR-attributable losses over time. For the global non-water total, fitted absolute-loss slopes range from 1.75 billion Int$2020 $yr^{-2}$ under SSP3-7.0 to 4.66 billion Int$2020 $yr^{-2}$ under SSP5-8.5. Across the global and ecosystem-specific series, $R^2$ values range from 0.857 to 0.987 for absolute losses and from 0.958 to approximately 1.000 for relative losses. The fitted lines and 95% confidence bands summarize long-term trends rather than implying that losses at individual time points follow a strictly linear process. Because the monetary estimates combine SSP-specific SLR exposure and SSP-dependent income-adjusted unit values, their ordering among pathways should not be interpreted as a simple emissions gradient.Figure S3 shows the signed net nine-class ESV accounting result obtained by adding the water-body loss values to the global non-water ESV losses used in the main analysis under five SSP pathways from 2030 to 2100. Panel (a) presents the calculated trajectories, whereas panel (b) presents the corresponding ordinary least-squares linear fits with 95% confidence bands. The net accounting result is negative at every time point and becomes increasingly negative toward 2100, ranging from −129.24 billion Int$2020 $yr^{-1}$ under SSP3-7.0 to −342.44 billion Int$2020 $yr^{-1}$ under SSP5-8.5 in 2100. These negative values do not represent genuine gains in ecosystem services or compensation for losses of non-water ecosystems. Instead, they arise because areas inundated by SLR are reassigned from their original land-cover classes to water, causing the negative water-loss value to exceed the positive loss summed across the eight non-water classes. This accounting comparison therefore supports the exclusion of water from the global loss totals and all subsequent national-loss and injustice analyses.

### Cross-ecosystem differences in relative and absolute losses

Figures S4 and S5 extend the ecosystem-specific results in Fig. 3 to the four SSP pathways other than SSP2-4.5. Across SSP1-1.9, SSP1-2.6, SSP3-7.0, and SSP5-8.5, wetlands consistently experience the greatest relative ESV losses at all four selected time points (Fig. S4). In contrast, the composition of absolute ESV loss is dominated by impervious surfaces and croplands, which together account for 57.8–60.5% of the global non-water total across the four pathways and selected years (Fig. S5). These results confirm that the ecosystem type experiencing the greatest proportional loss is not necessarily the largest contributor to total monetary loss.

### National patterns of ecosystem-specific ESV losses

Figure S6 shows pronounced geographic inequality in wetland ESV losses under SSP2-4.5 in 2100. Absolute losses are concentrated in a limited number of countries, led by Russia (3.70 billion Int$2020 $yr^{-1}$), India (2.42 billion Int$2020 $yr^{-1}$), Romania (1.74 billion Int$2020 $yr^{-1}$), Argentina (1.71 billion Int$2020 $yr^{-1}$), and Brazil (1.47 billion Int$2020 $yr^{-1}$). In contrast, the largest relative wetland losses occur in Romania (44.84%), Benin (26.71%), Saint Lucia (22.22%), Libya (12.92%), and Egypt (11.99%). Romania therefore exhibits a notable dual pattern, ranking third in absolute wetland ESV loss and first in relative wetland ESV loss. Figure S7 compares national absolute ESV losses for impervious surfaces and croplands under SSP2-4.5 in 2100. China records the largest loss for both ecosystem types, reaching 21.99 billion Int$2020 $yr^{-1}$ for impervious surfaces and 12.78 billion Int$2020 $yr^{-1}$ for croplands. Impervious-surface losses are followed by Iraq (4.58 billion Int$2020 $yr^{-1}$), Egypt (1.90 billion Int$2020 $yr^{-1}$), Viet Nam (1.51 billion Int$2020 $yr^{-1}$), and Italy (1.35 billion Int$2020 $yr^{-1}$). Cropland losses are followed by Myanmar (9.40 billion Int$2020 $yr^{-1}$), Viet Nam (5.65 billion Int$2020 $yr^{-1}$), Egypt (4.95 billion Int$2020 $yr^{-1}$), and India (1.74 billion Int$2020 $yr^{-1}$). These results show that China is the dominant national hotspot for both ecosystem types, whereas their remaining loss distributions differ substantially across countries.

### Spatial variation in unit ESV coefficients

The 2024 baseline country–ecosystem unit values (unit ESV, Int\$2020 $ha^{-1}$ $yr^{-1}$) exhibit substantial spatial and ecosystem-specific variation (Figure S8a). Among ecosystem types, impervious surfaces display the highest mean unit ESV, followed by wetlands and water bodies, then forests, croplands, grasslands, and shrublands, whereas tundra and bare land generally have lower values (Figure S8b). For croplands, Ethiopia has the highest assigned unit ESV, reaching 7,328 Int\$2020 $ha^{-1}$ $yr^{-1}$. For forests, China has the highest assigned unit ESV, reaching 10,758 Int\$2020 $ha^{-1}$ $yr^{-1}$. Grassland and shrubland unit ESVs are relatively high in several European countries. Wetland unit ESV reaches its maximum in Oceania, exemplified by Palau at 20,063 Int\$2020 $ha^{-1}$ $yr^{-1}$, whereas water bodies are comparatively high in Asia, with China at 5,524 Int\$2020 $ha^{-1}$ $yr^{-1}$. Tundra and bare land generally present lower unit values, although tundra in northern Europe and North America shows slightly elevated values, and bare land in Europe exhibits higher ESV than in Asia or Oceania. These country-level differences reflect the eligible ESVD observations and hierarchical mean-assignment procedure and should not be interpreted as direct tests of climatic, ecological, or land-management mechanisms.

The composition of unit ESV further indicates that the dominant service category differs across ecosystem types. Provisioning services account for relatively large shares in croplands, water bodies, and bare land, whereas habitat services make the largest contribution to forest unit ESV and also contribute to wetland and water unit ESV. These spatial and ecosystem-specific patterns provide a consistent baseline for estimating total ESV losses under projected SLR scenarios. The income-adjustment factor equals 1 in 2024, and future SSP-dependent unit ESVs are calculated from this common baseline.

### Land-cover to ESVD classification crosswalk

Table S1 summarises the harmonised crosswalk used in this study to ensure consistent ESV accounting across datasets. It links classes in the 10-m global land-cover product (including level-2 subclasses) to the corresponding ESVD v2.0 biome and eco-zone categories, forming the basis for converting land-cover areas (by SSP, year, and country) into comparable ESV estimates.

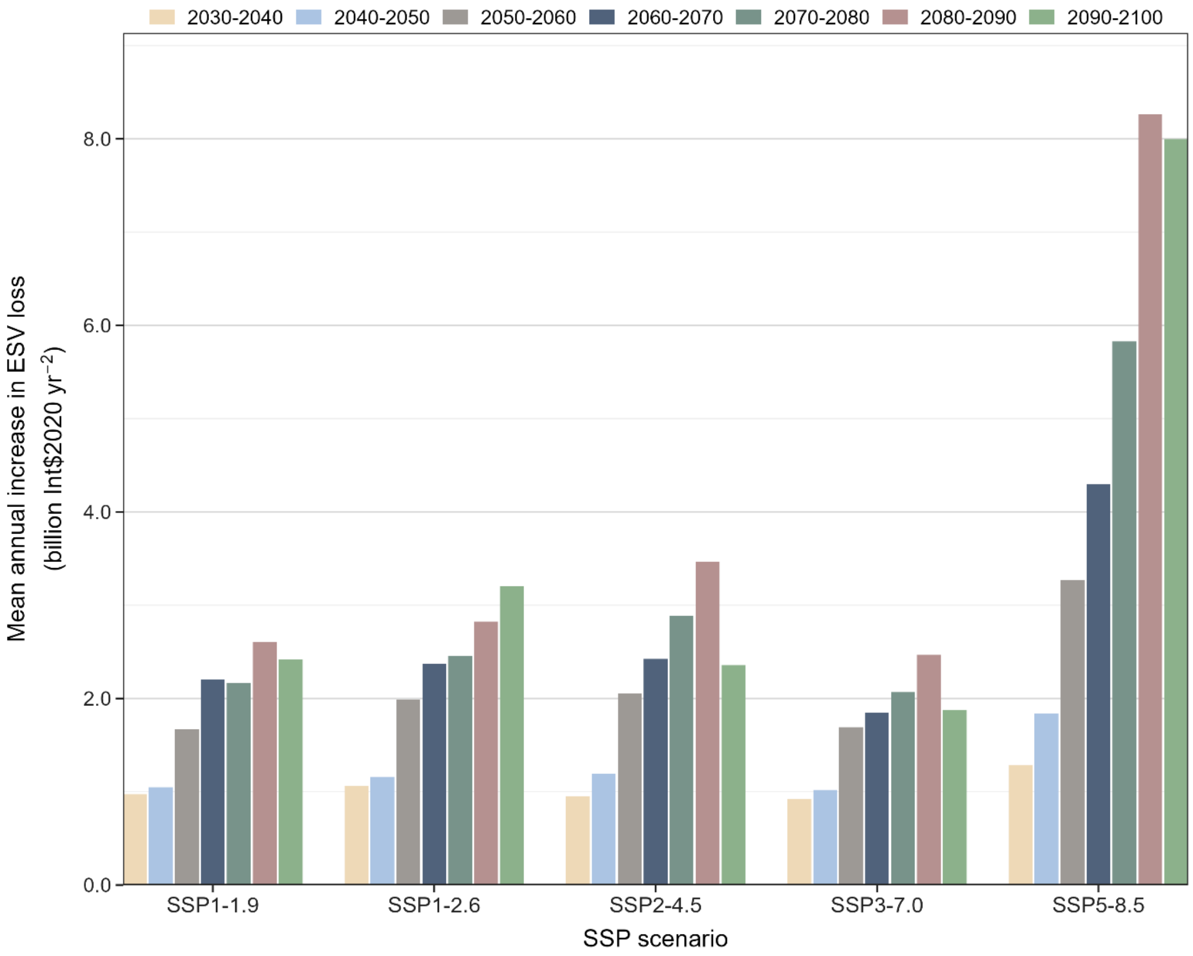


**Fig. S1. Mean annual increases in global income-adjusted absolute ESV loss attributable to SLR, excluding water bodies, across successive decadal intervals from 2030 to 2100 under five SSP pathways.** Bars represent the change in annual absolute ESV loss between the end and start of each interval divided by the interval length, expressed in billion Int$2020 $yr^{-2}$, with colors indicating time intervals. Positive values indicate that annual ESV losses increase between successive time points. The 2024–2030 interval is omitted because it spans six rather than ten years. Baseline unit ESV coefficients are from ESVD v2.0(1), SSP-dependent income adjustments follow the income trajectories described in the main-text Methods, and SLR projections are from IPCC AR6 (FACTS)(2–4).

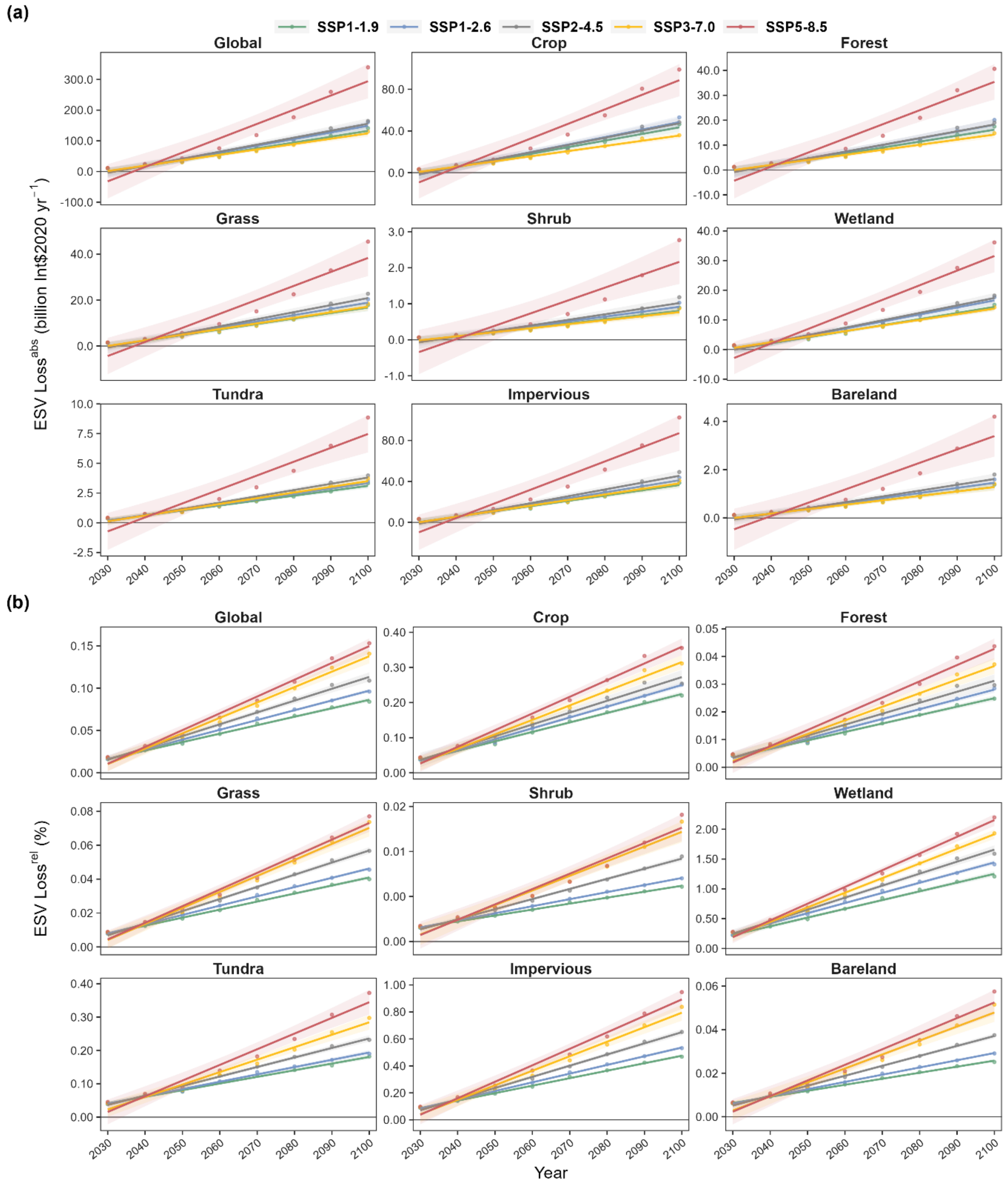


**Fig. S2. Linear fits of income-adjusted absolute and relative ESV losses attributable to SLR under five SSP pathways from 2030 to 2100.** (a) Absolute ESV losses (Loss$^{abs}$; billion Int$2020 yr$^{-1}$) for the global non-water total and eight non-water ecosystem types. (b) Corresponding relative ESV losses (Loss$^{rel}$; %), calculated relative to the scenario- and year-specific no-SLR counterfactual ESV. Points represent the estimates at eight decadal time points, solid lines represent ordinary least-squares linear fits, and shaded bands indicate 95% confidence intervals. The eight ecosystem types are cropland, forest, grassland, shrubland, wetland, tundra, impervious surface, and bare land. Water is excluded because its apparent gain represents the reclassification of inundated land as water. Baseline unit ESV coefficients are from ESVD v2.0**(1)**, SSP-dependent income adjustments follow the income trajectories described in the main-text Methods, and SLR projections are from IPCC AR6 (FACTS)**(2–4)**.

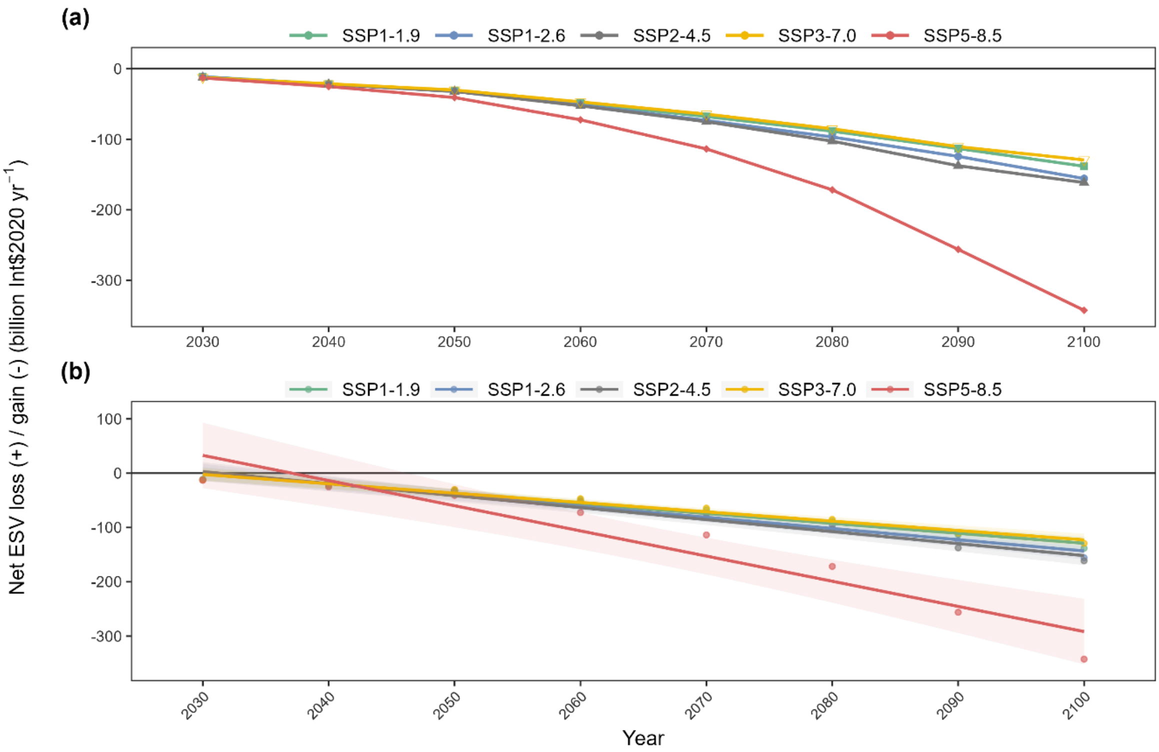


**Fig. S3. Signed net nine-class ESV accounting result under five SSP pathways from 2030 to 2100.** The net result combines the positive ESV losses across the eight non-water ecosystem classes with the negative water-loss value generated when inundated terrestrial areas are reclassified as water. (a) Calculated trajectories of the signed net accounting result. (b) Corresponding ordinary least-squares linear fits, with shaded bands indicating 95% confidence intervals. The vertical axis is expressed as net ESV loss (+) or gain (−) in billion Int$2020 $yr^{-1}$. Negative values indicate that the water accounting gain exceeds the summed losses across the eight non-water classes; they do not represent a genuine gain in ecosystem services or ecological compensation for losses of non-water ecosystems. Baseline unit ESV coefficients are from ESVD v2.0**(1)**, SSP-dependent income adjustments follow the income trajectories described in the main-text Methods, and SLR projections are from IPCC AR6 (FACTS)**(2–4)**.

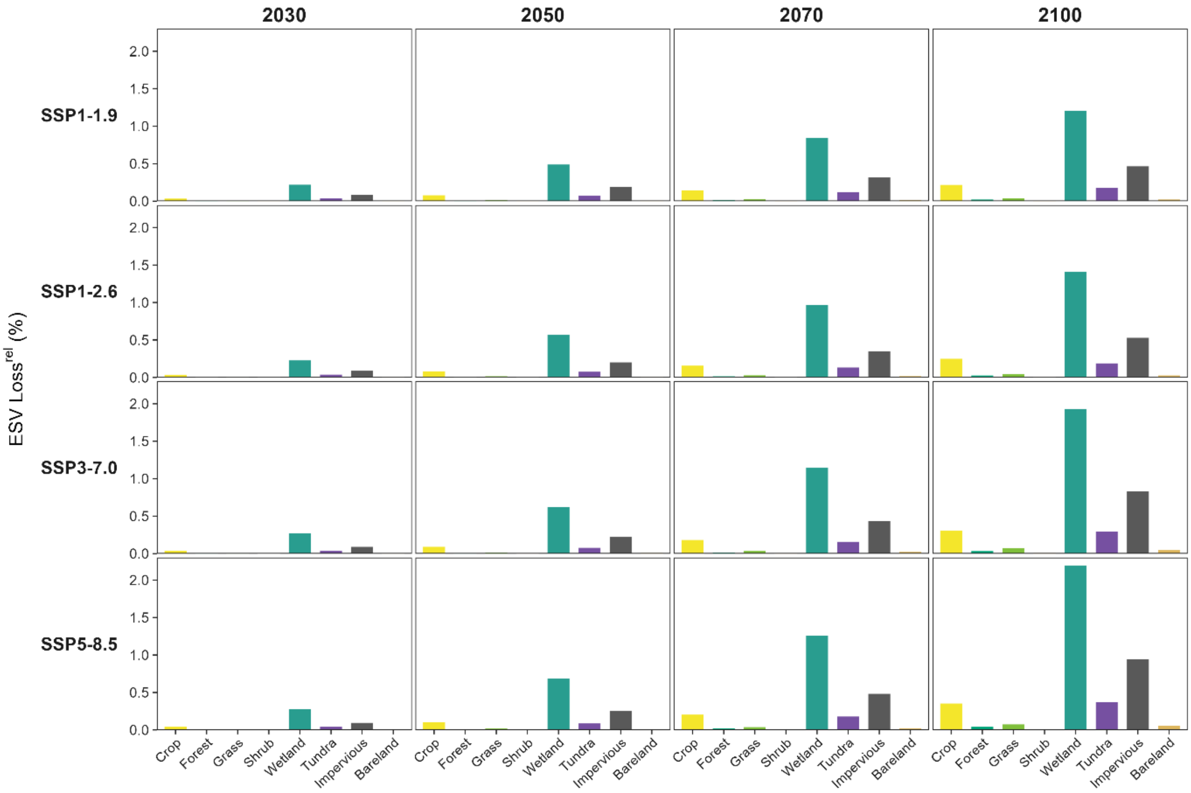


**Fig. S4. Relative ESV losses across eight non-water ecosystem types under the four remaining SSP pathways (SSP1-1.9, SSP1-2.6, SSP3-7.0, and SSP5-8.5).** Bars show relative ESV losses in 2030, 2050, 2070, and 2100. Rows represent SSP pathways, columns represent years, and colors denote ecosystem types. Relative losses are calculated against the corresponding scenario- and year-specific no-SLR counterfactual ESV, and positive values indicate losses. The eight ecosystem types are cropland, forest, grassland, shrubland, wetland, tundra, impervious surface, and bare land. Water is excluded. Baseline unit ESV coefficients are from ESVD v2.0**(1)**, SSP-dependent income adjustments follow the income trajectories described in the main-text Methods, and SLR projections are from IPCC AR6 (FACTS) **(2–4)**.

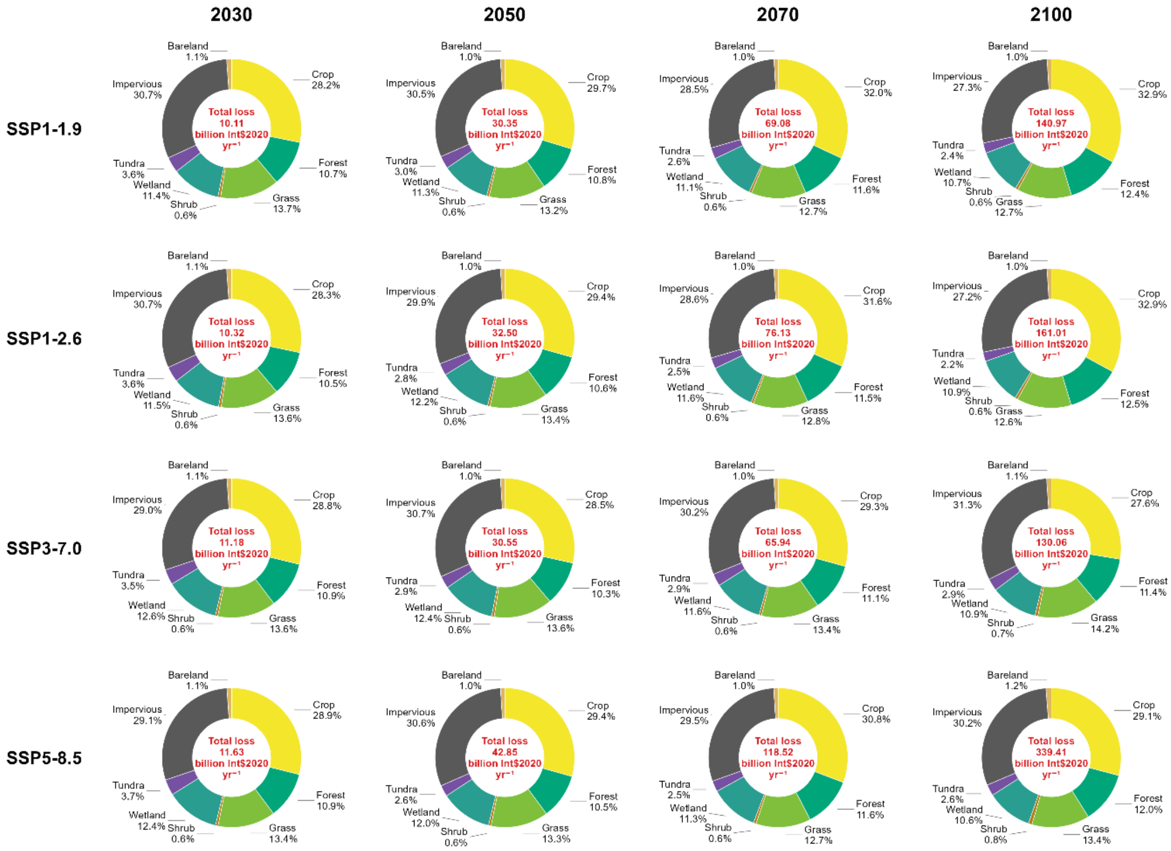


**Fig. S5. Composition of income-adjusted absolute ESV losses across eight non-water ecosystem types under the four remaining SSP pathways (SSP1-1.9, SSP1-2.6, SSP3-7.0, and SSP5-8.5).** Donut charts show the ecosystem-specific percentage contributions to global absolute ESV loss in 2030, 2050, 2070, and 2100 under SSP1-1.9, SSP1-2.6, SSP3-7.0, and SSP5-8.5. Rows represent SSP pathways and columns represent years. Values at the center of each donut indicate the corresponding global non-water total loss (billion Int$2020 $yr^{-1}$). Colors denote ecosystem types. The eight ecosystem types are cropland, forest, grassland, shrubland, wetland, tundra, impervious surface, and bare land. Water is excluded. Baseline unit ESV coefficients are from ESVD v2.0**(1)**, SSP-dependent income adjustments follow the income trajectories described in the main-text Methods, and SLR projections are from IPCC AR6 (FACTS) **(2–4)**.

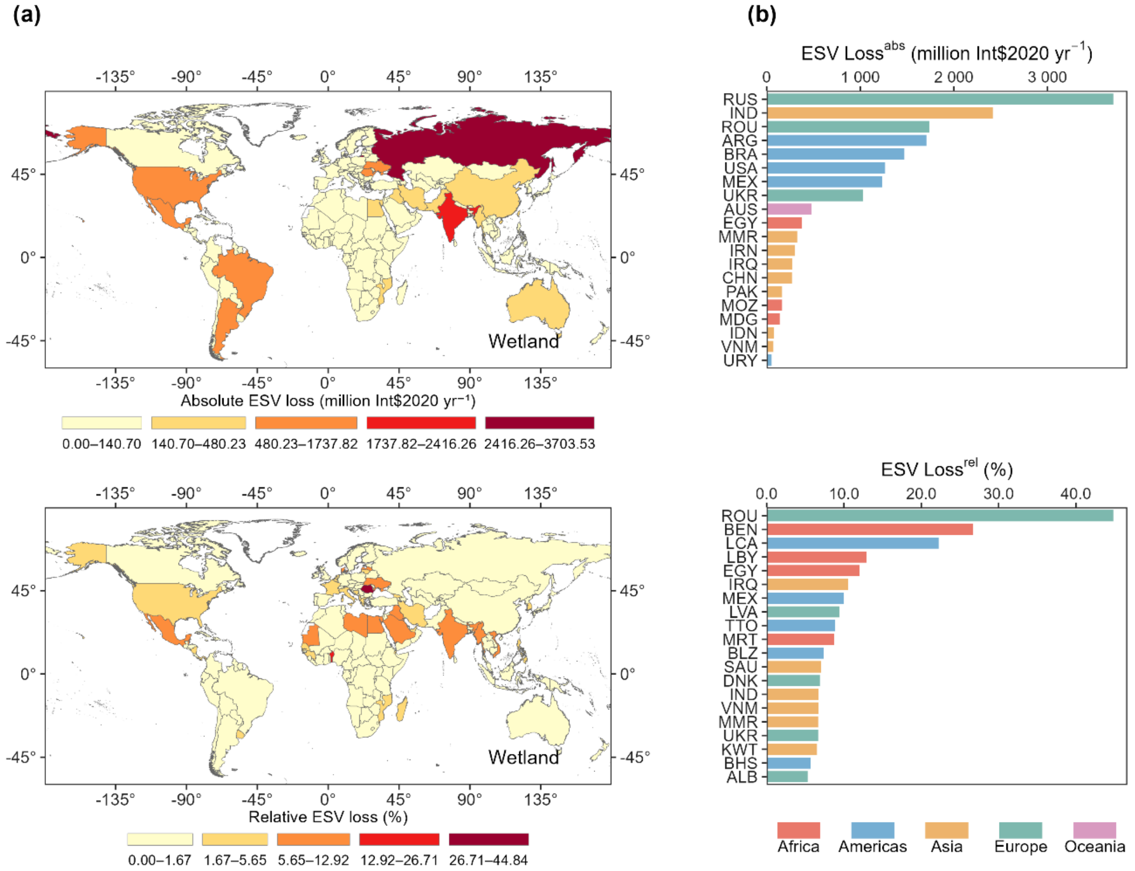


**Fig. S6. National patterns of income-adjusted wetland ESV losses attributable to SLR under SSP2-4.5 in 2100.** (a) Global maps of absolute wetland ESV loss (top; million Int$2020 yr$^{-1}$) and relative wetland ESV loss (bottom; %). (b) Top 20 countries ranked by absolute wetland ESV loss (top) and relative wetland ESV loss (bottom); bar colors indicate continents. Relative loss is calculated against each country's corresponding no-SLR wetland ESV, and positive values indicate losses. The analysis uses the consistent 171-country income-adjusted sample; countries without complete SSP GDP-per-capita data are shown in grey. Baseline unit ESV coefficients are from ESVD v2.0**(1)**, SSP-dependent income adjustments follow the income trajectories described in the main-text Methods, and SLR projections are from IPCC AR6 (FACTS) **(2–4)**.

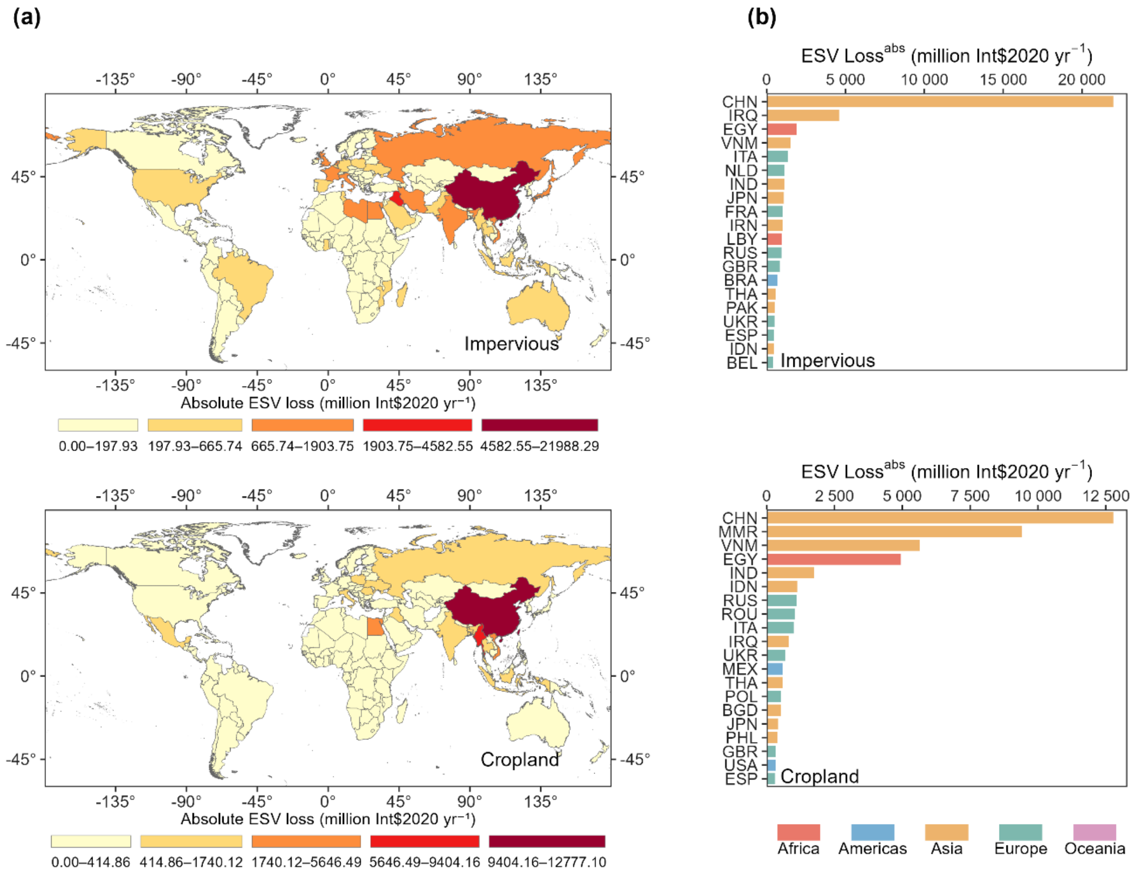


**Fig. S7. National distribution of income-adjusted absolute ESV losses for impervious surfaces and croplands under SSP2-4.5 in 2100.** (a) Global maps of national absolute ESV loss (million Int$2020 $yr^{-1}$) for impervious surfaces (top) and croplands (bottom). (b) Top 20 countries ranked by absolute ESV loss for impervious surfaces (top) and croplands (bottom); bar colors indicate continents. Positive values indicate losses. The analysis uses the consistent 171-country income-adjusted sample; countries without complete SSP GDP-per-capita data are shown in grey. Baseline unit ESV coefficients are from ESVD v2.0**(1)**, SSP-dependent income adjustments follow the income trajectories described in the main-text Methods, and SLR projections are from IPCC AR6 (FACTS) **(2–4)**.

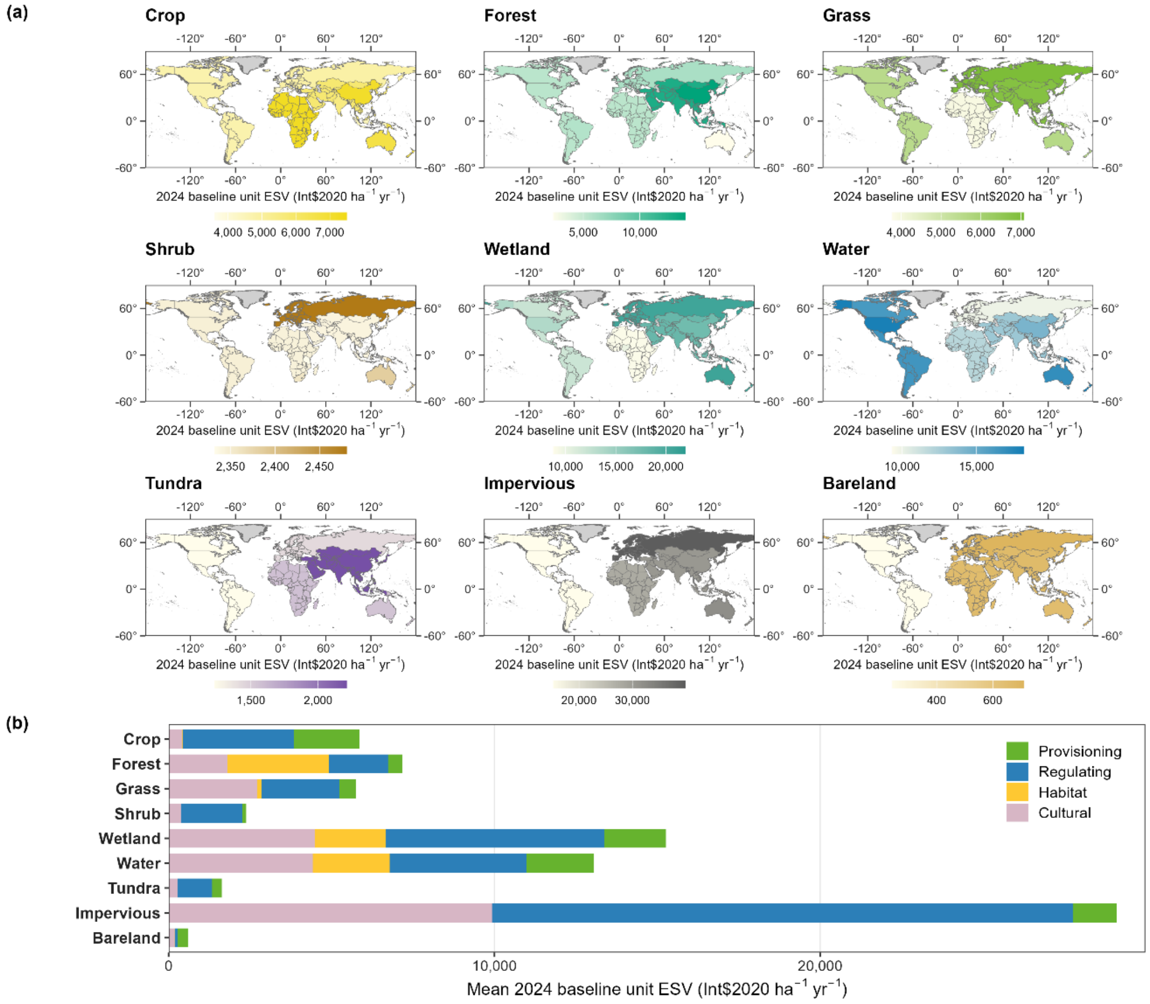


**Fig. S8. Spatial distribution and composition of 2024 baseline unit ESV across nine ecosystem types.** (a) Country-level spatial distribution of 2024 baseline unit ESV (Int$2020 $ha^{-1}$ $yr^{-1}$) for nine ecosystem types: cropland, forest, grassland, shrubland, wetland, water, tundra, bare land, and impervious surface. (b) Mean 2024 baseline unit ESV and the relative contribution of four ecosystem service categories—provisioning, regulating, habitat, and cultural—across ecosystem types. The income-adjustment factor equals 1 in 2024; future SSP-dependent unit ESVs are calculated from these baseline coefficients. ESV data are from ESVD v2.0**(1)**.

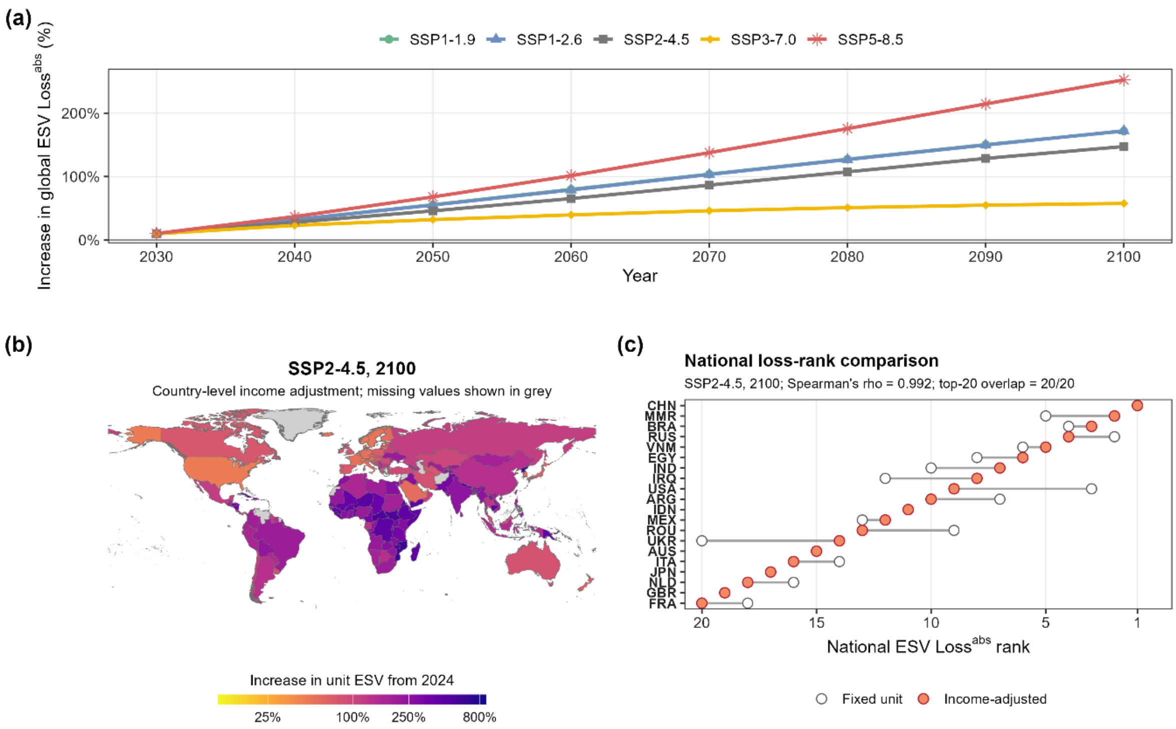


**Fig. S9. Effects of SSP-dependent income adjustment on unit ecosystem service values and projected ESV losses attributable to sea-level rise.** (a) Percentage increase in global annual absolute ESV loss estimated using income-adjusted unit values relative to the corresponding estimate based on fixed 2024 unit values from 2030 to 2100 under the five SSPs. Both estimates use the same SLR-induced ecosystem-area changes, exclude water, and are calculated over the same 171-country sample. (b) Country-level percentage increase in unit ESV from the 2024 baseline under SSP2-4.5 in 2100, calculated as ($F_{c,s,t}$ - 1) × 100%, where $F_{c,s,t}$ is the country-, scenario-, and year-specific income-adjustment factor. The same percentage adjustment is applied to all ecosystem types within a country. Countries without available SSP GDP-per-capita data are shown in grey. (c) Comparison of national absolute ESV-loss ranks under fixed-unit and income-adjusted valuation for SSP2-4.5 in 2100 among the 171 countries included in the income-adjusted loss analysis. Open and filled circles indicate fixed-unit and income-adjusted ranks, respectively, and grey segments connect estimates for the same country. Rank 1 represents the largest national absolute loss. National rankings remain strongly correlated (Spearman's $\rho$ = 0.992), and the two valuation approaches identify the same 20 highest-loss countries.

**Table S1. Crosswalk between ESVD 2.0 Classification and Land Cover Taxonomy.**

| Land Cover | Land Cover level 2 | ESVD 2.0 Biome | ESVD 2.0 Ecozone |
|---|---|---|---|
| 10Crop | Rice 10/11, Greenhouse 10/12, Other 10/13 | 13 Intensive land use | |
| 20Forest | Broadleaf 20/21, Needleleaf 20/22, Mixed 20/23, Orchard 20/24 | 5 Tropical and subtropical forests; 6 Temperate forest and woodland; 7 Cold climate evergreen forest and woodland | |
| 30Grass | Managed 30/31, Nature 30/32 | 9 Rangelands and natural grasslands | |
| 40Shrub | | 8 Shrubland and shrubby woodland | |
| 50Wetland | Grass 30/51, Silt 90/52 | 3 Inland wetlands | 2.4 Coastal wetlands and river deltas |
| 60Water | Lake 60/61, Pond 60/62, River 60/63, Sea 60/64 | 1 Marine; 4 Rivers and lakes | 2.3 Coastal inlets |
| 70Tundra | Shrub 40/71, Grass 30/72 | | 11.2 Polar alpine rocky outcrops, 11.3 Polar tundra and deserts, 11.4 Temperate alpine meadows and shrublands, 11.5 Tropical alpine grasslands and herb fields |
| 80Impervious | High albedo 80/81, Low albedo 80/82 | 14 Anthropogenic systems; 15 Urban green and blue infrastructure | |
| 90Bareland | Saline-Alkali 90/91, Sand 90/92, Gravel 90/93, Bare-cropland 10/94, Dry river/lake bed 90/95, other 90/96 | 10 Desert and semi desert | 2.1 Shorelines, 2.2 Coastal dunes and cliffs |
| 100Snow/Ice | Snow 100/101, Ice 100/102 | | 11.1 Ice sheets, glaciers and perennial snowfields |

**Note**: Land-cover classes are derived from the 10-m global land-cover product developed by Gong et al.(5, 6). The ESVD v2.0 biome–ecosystem classification(1) is based on the FAO Global Ecological Zones(7) and the IUCN Global Ecosystem Typology(8). The crosswalk was constructed by harmonizing land-cover legend categories with the corresponding ESVD biome and ecozone classes.

**Dataset S1 (separate file).** Processed valuation database and 2024 country–ecosystem unit ecosystem service value (unit ESV) coefficients. The dataset contains total unit ESV coefficients for 1,737 country–ecosystem combinations covering 193 United Nations member states and nine ecosystem classes, together with service-level transferred-value records. For each country–ecosystem–service combination, it reports the applied unit value, whether the value was derived from country-level, continental, or global evidence, the source geography, the number of eligible valuation observations, and the number of distinct primary studies. Monetary values are expressed in international dollars per hectare per year at 2020 price levels (Int\$2020 $ha^{-1}$ $yr^{-1}$).

**Dataset S2 (separate file).** Country- and global-level ESV projections, SLR-attributable losses, and fitted-equation results. The dataset reports income-adjusted and fixed-unit-value absolute and relative ESV losses under five SSP pathways from 2030 to 2100 for the consistent 171-country sample. It also provides the regression results underlying Figs. S2 and S3 and the corresponding country-level loss fits, including the slope, intercept, fitted value in 2030, equation centered at 2030, $R^2$, RMSE, number of time points, and fitting period. Monetary losses are expressed in international dollars at 2020 price levels (Int\$2020 $yr^{-1}$), and relative losses are calculated against the corresponding scenario- and year-specific no-SLR counterfactual ESV.

## SI References


1. Brander, L. M., *et al.*, Ecosystem Services Valuation Database (ESVD). Deposited 2025.

2. R. E. Kopp, *et al.*, The Framework for Assessing Changes To Sea-level (FACTS) v1.0: a platform for characterizing parametric and structural uncertainty in future global, relative, and extreme sea-level change. *Geoscientific Model Development* **16**, 7461–7489 (2023).

3. Intergovernmental Panel on Climate Change (IPCC), Ed., "Ocean, Cryosphere and Sea Level Change" in *Climate Change 2021 – The Physical Science Basis: Working Group I Contribution to the Sixth Assessment Report of the Intergovernmental Panel on Climate Change*, (Cambridge University Press, 2023), pp. 1211–1362.

4. G. G. Garner, *et al.*, IPCC AR6 Sea Level Projections. Zenodo. https://doi.org/10.5281/zenodo.6382554. Deposited 9 August 2021.

5. P. Gong, *et al.*, Stable classification with limited sample: transferring a 30-m resolution sample set collected in 2015 to mapping 10-m resolution global land cover in 2017. *Science Bulletin* **64**, 370–373 (2019).

6. P. Gong, J. Wang, H. Huang, Stable classification with limited samples in global land cover mapping: Theory and experiments. *Sci Bull (Beijing)* **69**, 1862–1865 (2024).

7. FAO, Global ecological Zones for FAO forest reporting: 2010 update. (2012).

8. D. A. Keith, J. R. Ferrer-Paris, E. Nicholson, R. T. Kingsford, Eds., *IUCN Global Ecosystem Typology 2.0: descriptive profiles for biomes and ecosystem functional groups* (IUCN, International Union for Conservation of Nature, 2020).
